# Supplementary material for: Light-Activated Liposomes Coated with Hyaluronic Acid as a Potential Drug Delivery System
Source: Pharmaceutics. 2020 Aug 12;12(8):763. doi: 10.3390/pharmaceutics12080763 (PMC7465487; doi:10.3390/pharmaceutics12080763)

**Supplementary Information**

Hyaluronic acid-coated light-activated liposomes: synthesis, stability, drug release, vitreal mobility and corona formation in plasma and vitreous

Otto K. Kari ^1^, Shirin Tavakoli ^1^, Petteri Parkkila ^1^, Simone Baan ^1,2^, Roosa Savolainen ^1^, Teemu Ruoslahti ^1^, Niklas G. Johansson ^3^, Joseph Ndika ^4^, Harri Alenius ^4,5^, Tapani Viitala ^1,3^, Arto Urtti ^1,6,7^ and Tatu Lajunen ^1,8,^*

^1^ Drug Research Program, Division of Pharmaceutical Biosciences, Faculty of Pharmacy, University of Helsinki, Viikinkaari 5 E, FI-00790 Helsinki, Finland; otto.kari@helsinki.fi (O.K.K.); shirin.tavakoli@helsinki.fi (S.T.); petteri.parkkila@helsinki.fi (P.P.); s.d.baan@uu.nl (S.B.); roosa.pp.savolainen@helsinki.fi (R.S.); teemu.ruoslahti@aalto.fi (T.R.); tapani.viitala@helsinki.fi (T.V.); arto.urtti@helsinki.fi (A.U.)

^2^ Pharmaceutics, Utrecht Institute for Pharmaceutical Sciences, Utrecht University, P.O. Box 80.082, 3508 TB Utrecht, The Netherlands

^3^ Drug Research Program, Division of Pharmaceutical Chemistry and Technology, Faculty of Pharmacy, University of Helsinki, Viikinkaari 5 E, FI-00790 Helsinki, Finland; niklas.johansson@helsinki.fi (N.G.J.)

^4^ Human Microbiome Research, Faculty of Medicine, University of Helsinki, Haartmaninkatu 3, FI-00290 Helsinki, Finland; joseph.ndika@helsinki.fi (J.N.); harri.alenius@helsinki.fi (H.A.)

^5^ Institute of Environmental Medicine, Karolinska Institutet, 171 77 Stockholm, Sweden

^6^ School of Pharmacy, Faculty of Health Sciences, University of Eastern Finland, Yliopistonranta 1, 70210 Kuopio, Finland

^7^ Institute of Chemistry, St. Petersburg State University, Petergof, Universitetskii pr. 26, 198504 St. Petersburg, Russia

^8^ Laboratory of Pharmaceutical Technology, Department of Pharmaceutical Science, Tokyo University of Pharmacy & Life Sciences, 1432-1 Hachioji 192-0392, Tokyo, Japan

***** Correspondence: tatu.lajunen@helsinki.fi (T.L.); Tel.: +81-7044-589-511

**Figure S1** _1_H NMR and FT-IR spectra of the synthetized DSPE-HA conjugate ............................................... 2

**Figure S2.** Size, T_m_ profiles, heat and light-triggered calcein releases and ICG absorbances ........................ 3

**Figure S3.** Size, T_m_ profiles, heat-triggered calcein releases and ICG absorbances ........................................ 4

**Figure S4.** Dynamic light scattering and large angle light scattering .............................................................. 5

**Figure S5.** Heatmap of the 178 proteins identified in human plasma ............................................................... 6

**Figure S6.** Heatmap of the 728 proteins identified in porcine vitreous ............................................................ 7

**Figure S7.** Properties of the Top 20 enriched proteins ..................................................................................... 8

**Figure S8.** Enriched functions and gene interaction network of HC proteins only enriched on HA-coated

liposomes in plasma ......................................................................................................................................... 11

**Figure S9.** Enriched biological functions and gene interaction network based on top 20 most abundant HC

proteins in plasma. ........................................................................................................................................... 14

**Figure S10.** Enriched biological functions and gene interaction network based on top 20 most abundant HC

proteins in vitreous ........................................................................................................................................... 16

**Table S1.** Venn diagram of the protein-encoding genes in the plasma HC intersection ................................... 8

**Table S2.** Venn diagram of the protein-encoding genes in the vitreous HC union ........................................... 9

**Table S3.** Enriched biological functions in the HC-enriched outlier sets per liposome group in plasma ........ 9

**Table S4.** Top 100 enriched biological functions of the top 20 most abundant HC proteins in plasma ......... 11

**Table S5.** Enriched biological functions based on the top 20 most abundant HC proteins in vitreous .......... 15


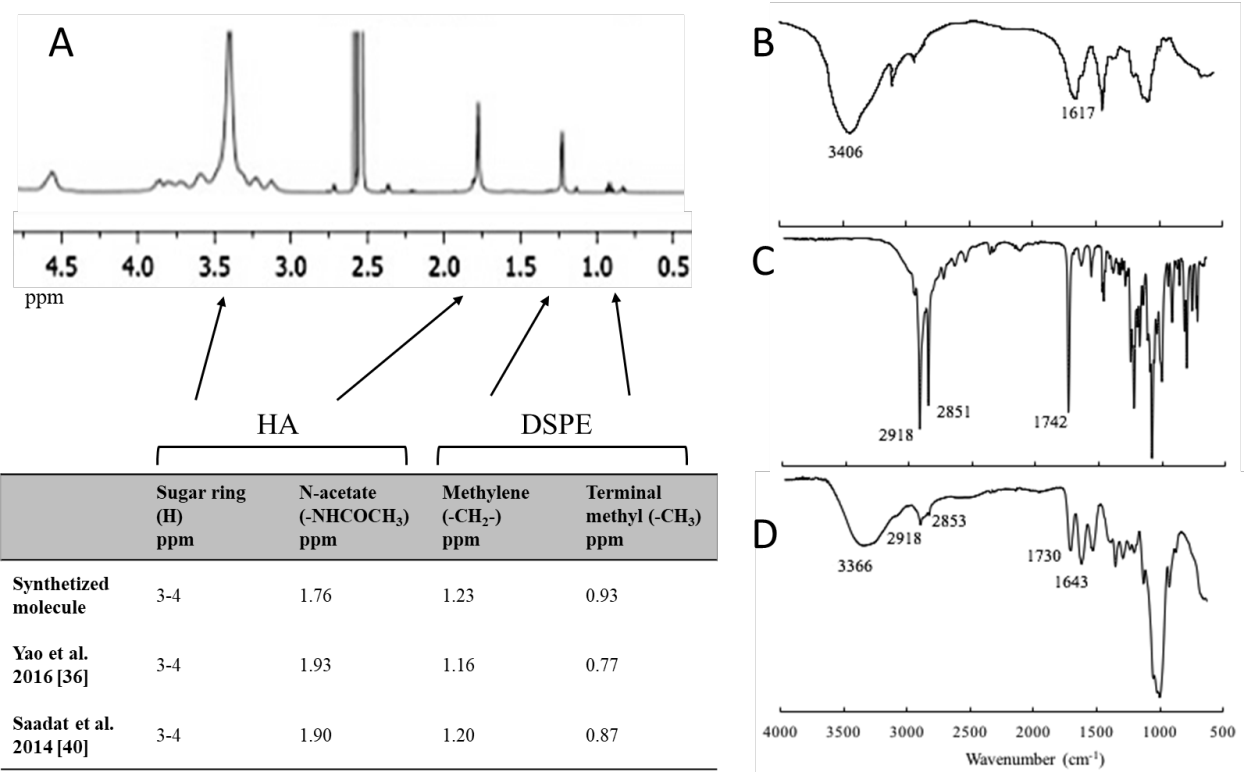


**Figure S1** **A.** _1_H NMR spectra of the synthetized DSPE-HA conjugate. The NMR spectra were in accordance with the previous literature, with the distinctive peaks at 3-4, 1.9, 1.2 and 0.9 ppm, indicating successful conjugation [1,2]. **B-D.** FT-IR spectra of HA (B), DSPE (C) and synthetized DSPE-HA molecule (D). Likewise, the FT-IR spectra showed the wide peak for the HA hydroxyl and amine groups (3200-3900 cm_-1_), the peak at for the carbonyl stretch of hyaluronic carboxylic acid (1643 cm_-1_), and the peaks for DSPE carbonyl groups (1730 cm_-1_) and acyl chains (2851–2918 cm_-1_) [1,3–5]. The spectra for HA is derived and modified from Yao et al. (2016) [36].


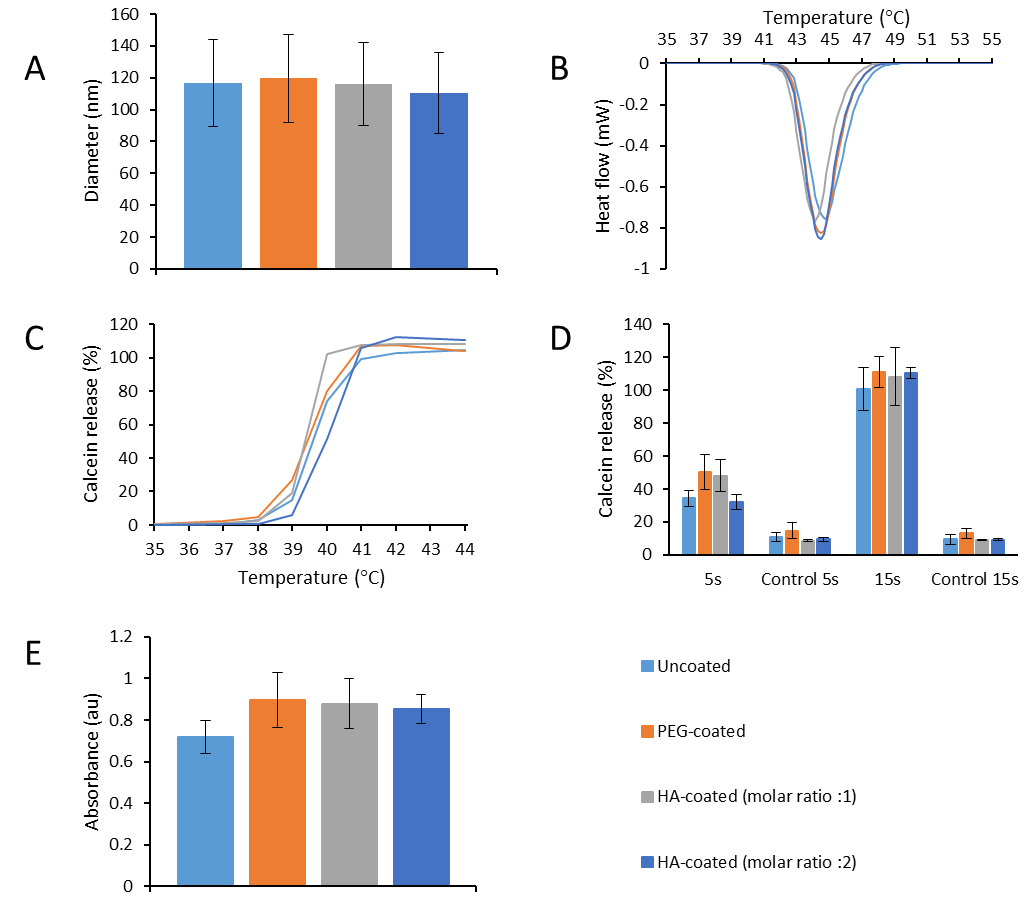


**Figure S2.** Size (**A**), T_m_ profiles (**B**), heat (**C**) and light-triggered (**D**) calcein releases and ICG absorbances (**E**) of uncoated (light blue), PEG-coated (orange) and hyaluronic acid (HA) coated liposomes with the HA molar ratio of 1 (grey) or 2 (dark blue). *N* = 3.


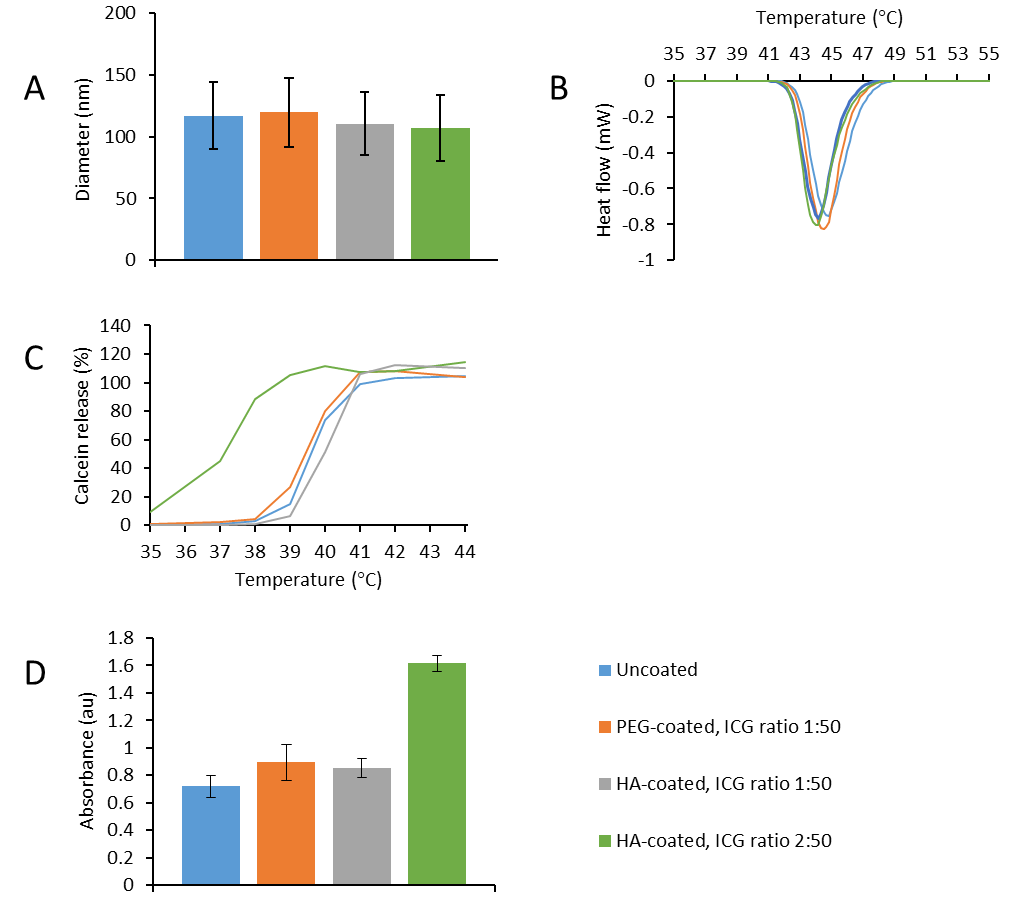


**Figure S3.** Size (**A**), T_m_ profiles (**B**), heat-triggered (**C**) calcein releases and ICG absorbances (**D**) of uncoated (light blue), PEG-coated (orange) and hyaluronic acid coated liposomes with the ICG to lipid molar ratio of 1:50 (grey) or 2:50 (green). *N* = 3.


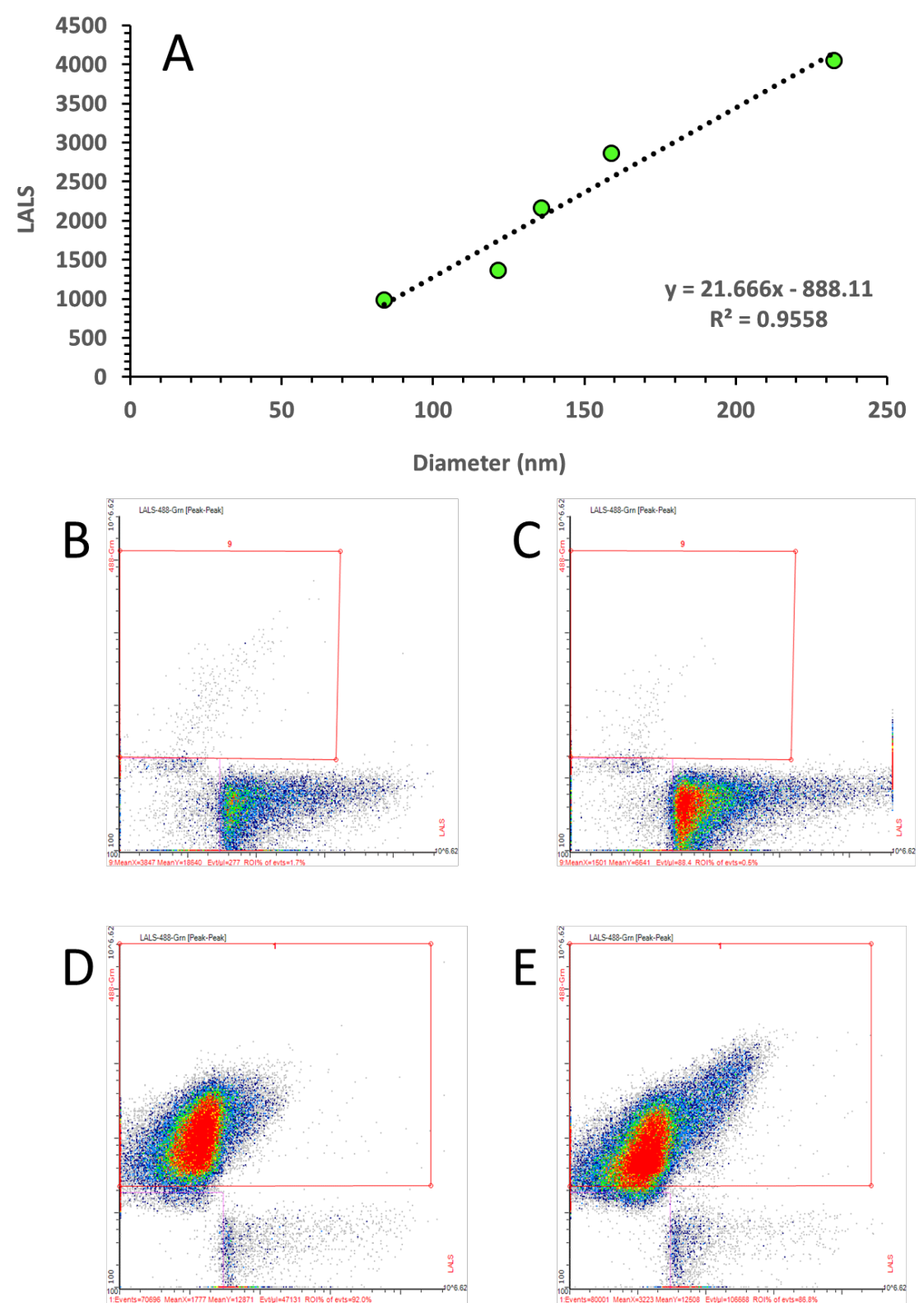


**Figure S4.** **A.** Calibration line of the size of polyethylene glycol (PEG) liposomes measured with dynamic light scattering (DLS) (size distribution by number in nm) and flow cytometry with Apogee Flow Systems (large angle light scattering (LALS), which results in a relative size). **B and C.** Apogee background noise with porcine vitreous (B) and human plasma (C). **D and E.** Representative graphs of HA-coated liposomes in porcine vitreous at 0-hour (D) and 48-hour time points (E). Red squares are the gated areas. X-axis is relative size of large angle light scattering (LALS) and y-axis is fluorescence, 488-Grn.


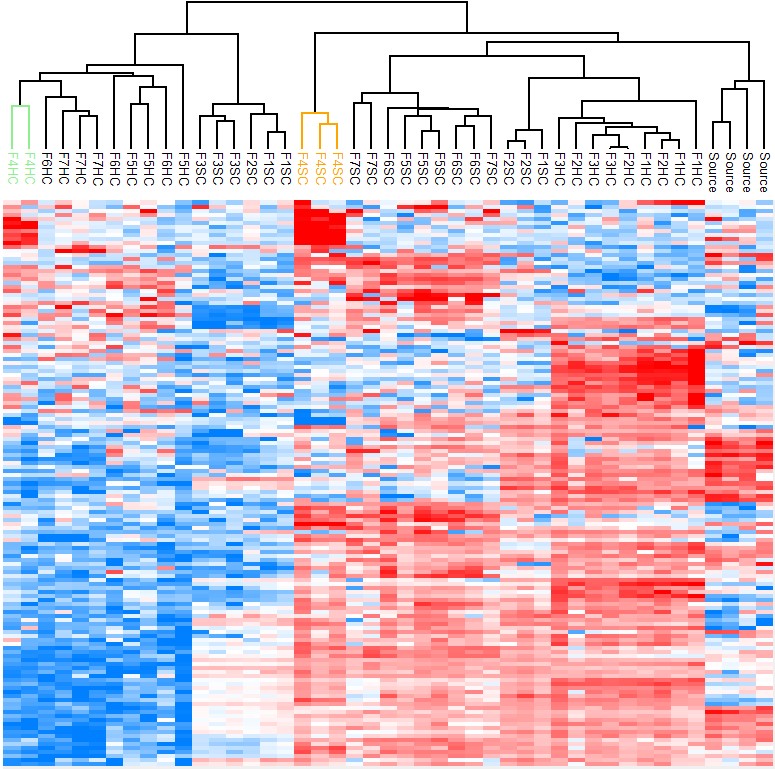


**Figure S5.** Heatmap of the 178 proteins identified in human plasma and their relative abundance after mediannormalization. Distinct clusters are observed for the HC, SC, and plasma source (including 1x and 7x diluted, SPR instrument flow-through, and untreated). The HC subsections of the tested formulations cluster based on their anionic or neutral surface charge. The range for relative enrichment (red) and depletion (blue) is two standard deviations from the mean on the log_2_ scale. The HC and SC replicates of the HA-coated liposome with ICG (F4) are highlighted.


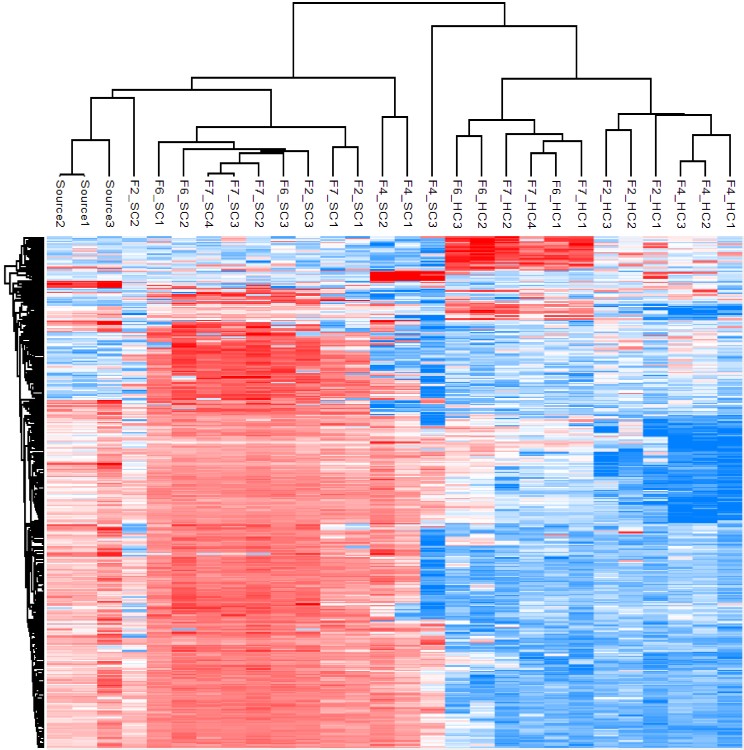


**Figure S6.** Heatmap of the 728 proteins identified in at least three samples and their relative abundance in porcine vitreous after median-normalization. Distinct clusters are observed for the HC, SC and plasma source. The HC subsections of the tested formulations do not cluster based on their anionic (F4, F6-F7) or neutral (F1F3) surface charge. The range for relative enrichment (red) and depletion (blue) is two standard deviations from the mean on the log_2_ scale.


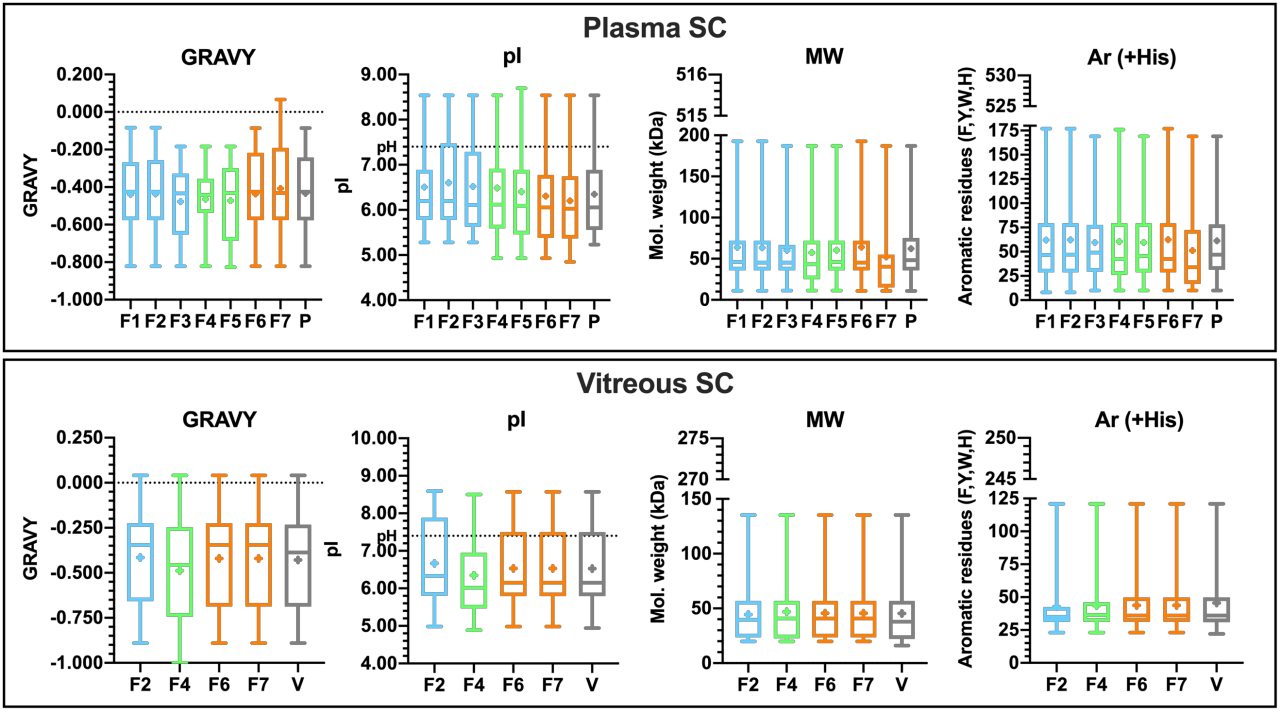


**Figure S7.** Properties of the Top 20 enriched proteins in the soft coronas (SC) and the source plasma or vitreous (P or V) with the max-min range, first and second quartiles, median, and mean (+). The color-coded groups are neutral 100 nm (blue), anionic 100 nm HA-coated (green) and small 50 nm anionic (orange) liposomes. GRAVY: grand average of hydropathy (positive score indicates hydrophobicity); pI: theoretical isoelectric point (pI under pH indicates net negative charge); MW: molecular weight; Ar (+His): aromatic residues phenylalanine, tyrosine, tryptophan and histidine.

| **Table S1.** Venn diagram of the protein-encoding genes in the **PLASMA HC** intersection between liposome groups. | | |
| --- | --- | --- |
| **Group** | **Number** | **Gene names** |
| F1 to F3  F4 and F5  F6 and F7 | 83 | PON1 ORM1 FGA AMBP AGT RBP4 IGHG4 LRG1 CFB FGG APCS ITIH1 C4BPA  C4A PLG IGHG1 IGHG2 A2M SERPINC1 CP SERPIND1 PGLYRP2 SERPING1  MAT2B ITIH4 IGLC2 IGHD F2 APOA1 HPR GC AZGP1 SERPINA1 CLU C1S C1QB  APOA2 C1R IGHM ORM2 AHSG C1QC PROS1 AFM IGKC TF HPX C8A IGHG3  APOC2 APOL1 APOD HRG LPA CD5L IGJ GSN C6 C5 APOC3 IGLL5 KNG1  SERPINA3 A1BG FN1 VTN C9 IGFALS SERPINF2 IGHA1 APOE APOA4 APOB  ITIH2 CFH TTR SERPINF1 KLKB1 C3 F12 APOH FGB HP |
| F1 to F3 F4 and F5 | 20 | CDH1 APOC1 CFI COLEC11 FBLN1 CFHR1 FGL1 ANGPTL6 MASP1 C8B APOA5 SERPINA6 VWF PLTP IGHA2 PF4 PRG4 C7 F11 F5 |
| F1 to F3 F6 and F7 | 3 | C8G APOM GAPDH |
| F4 and F5 F6 and F7 | 3 | SAA4 HBB IGHV3-23 |
| F1 to F3 | 3 | ENO1 CFP CPN2 |
| F4 and F5 | 4 | IGKV4-1 CRYAB LUM JCHAIN |
| F6 and F7 | 5 | CPN1 CRYBB2 AKAP1 GPLD1 ACTG1 |

| **Table S2.** Venn diagram of the protein-encoding genes in the **VITREOUS** **HC** union between liposome groups. | | |
| --- | --- | --- |
| **Group** | **Number** | **Gene names** |
| F2  F4  F6-F7 | 65 | GPX4 ENO1 GAPDHS SFRP2 ALB TUBB CFL1 HSPB1 CRYAB ETFA CKB HBZ  GNAT1 APOA1 GC CLU RLBP1 CRYBB2 HNRNPM GAPDH MDH2 LOC100739163  PFKM APOD CRYBB1 PCBP1 FBLN2 GSN CRYBB3 SPON1 RPS3 DPYSL2 LDHB  PKM TTR PTGDS HNRNPU CRYAA LTF RBP4 C4A DPYSL4 CST3 A2M CP SNCG  SAG SPP1 HSPA8 ETFB ALDOC TUBB1 RAN HNRNPK HBB RBP3 TUBA1B FN1 CRMP1 GNB1 PRPH APOE YWHAZ C3 GFAP |
| F2  F4 | 3 | PLG LOC100523213 RPS20 |
| F2  F6-F7 | 81 | ACTB GOT2 HSPA5 BFSP1 RTN4 CHGB CCT4 VCAN ZNF385A SNCA PRELP  GYG1 PPIA KPNB1 YWHAH KIF5B RAB3A MATR3 PROS1 SIRT2 CTSD MYEF2  BFSP2 VAT1 YWHAG CNRIP1 EFEMP2 CRABP2 CAPS GDI1 NSF ELN HSPA1B  GRIFIN HSP90AB1 ELAVL1 NRCAM LDHA CFB QSOX1 SH3GL1 PEBP1 STXBP1  COL9A2 SUCLA2 MAPK1 PHGDH NOVA1 EPB41L2 CLTC MAP2 TUBB4A GRK1  RPS13 RALY ENO2 AHSG HSPA12A RAB1A DPYSL3 ALDH1A2 HPX ARR3 MDH1  SOD1 RAB6A LOC110259374 RAB11B EFEMP1 LGALS1 UBE2V1 ABI3BP RAB7A  TPI1 PCSK1N DSTN PGK1 DHX9 RPL31 ATP5F1A DDX17 |
| F4  F6-F7 | 8 | PFKL RPS18 PCBP3 CRYBA4 TUBA1A F2 SERPINA1 STPG4 |
| F2 | 6 | CDHR1 HSP90AA1 HSP90A HSPCA LRR1 MAPT HNRNPC RPS3A LOC100155572 |
| F4 | 7 | FGA SERPINC1 PFKP NEFM ACTG1 FGB PCBP2 |
| F6-F7 | 85 | TPM3 HDGF ORM1 LCN2 PFN2 ATP4A RPS3A MAP4 DDAH1 IMPG1 ESD MAP1B  LMAN1 ASRGL1 UBE2NL HNRNPH1 COL18A1 AKR1A1 CLSTN1 DCTN2 MAP6  HRG ENPP2 UBB SERPINA3-2 RAB14 PPA1 TUFM A1BG TMSB4 GOT1 ILF3 PLK4  ATP5F1B ATP6V1B2 CFH ACOT7 MSI2 PRDX1 AKR1B1 SPARCL1 CDH2 PRDX6  CA2 ILF2 TKFC HSPG2 SRSF3 NME2 NCL KIAA0513 GUK1 GNGT1 CRYM PLD3 SERPIND1 CSTB CHGA SELENBP1 ITIH4 LAP3 IMPDH1 KHSRP UCHL1 HMGB2  AHCY FBN2 APP MIF EEF1G ATP6V1A PFN1 OXR1 APLP1 GNAT2 NSFL1C  HSP90AA1 FSCN1 HAPLN1 GNB3 BIN1 PRDX2 YWHAB VAMP1 HP |

| **Table S3.** Enriched biological functions in the HC-enriched *outlier* sets per liposome group in plasma. Highlighted functions in Fig SX in bold text. | | | | |
| --- | --- | --- | --- | --- |
| **Group** | **Genes** | **Function** | **FDR** | **Genes network/genome** |
| F1 to F3 | ENO1  CFP  CPN2 | **glycolysis** | 6,29E-17 | 10/48 |
|  |  | glucose catabolic process | 2,49E-16 | 10/58 |
|  |  | hexose catabolic process | 1,69E-15 | 10/72 |
|  |  | monosaccharide catabolic process | 1,70E-15 | 10/74 |
|  |  | single-organism carbohydrate catabolic process | 2,35E-14 | 10/97 |
|  |  | carbohydrate catabolic process | 3,30E-14 | 10/102 |
|  |  | gluconeogenesis | 1,34E-12 | 8/50 |
|  |  | hexose biosynthetic process | 1,64E-12 | 8/52 |
|  |  | monosaccharide biosynthetic process | 2,18E-12 | 8/55 |
|  |  | glucose metabolic process | 2,18E-12 | 10/161 |
|  |  | hexose metabolic process | 6,89E-12 | 10/182 |
|  |  | monosaccharide metabolic process | 2,56E-11 | 10/209 |
|  |  | regulation of protein activation cascade | 5,26E-10 | 6/27 |
|  |  | regulation of complement activation | 5,26E-10 | 6/27 |
|  |  | carbohydrate biosynthetic process | 7,58E-10 | 8/117 |
|  |  | regulation of humoral immune response | 2,08E-09 | 6/34 |
|  |  | complement activation | 8,82E-09 | 6/43 |
|  |  | regulation of acute inflammatory response | 1,28E-08 | 6/46 |
|  |  | humoral immune response | 2,62E-08 | 7/106 |
|  |  | protein activation cascade | 6,74E-08 | 6/61 |
|  |  | acute inflammatory response | 1,05E-07 | 6/66 |
|  |  | regulation of inflammatory response | 1,0181E-05 | 6/141 |
|  |  | inflammatory response | 2,0955E-05 | 7/283 |
|  |  | regulation of immune effector process | 4,5781E-05 | 6/184 |
|  |  | regulation of protein processing | 7,2097E-05 | 6/200 |
|  |  | positive regulation vascular endothelial growth factor production | 0,00071735 | 3/17 |
|  |  | regulation of vascular endothelial growth factor production | 0,00098285 | 3/19 |
|  |  | vascular endothelial growth factor production | 0,00111415 | 3/20 |
|  |  | isomerase activity | 0,07132273 | 3/79 |
| F4 and F5 | IGKV4-1  CRYAB  LUM  JCHAIN | extracellular matrix organization | 5,68E-03 | 6/292 |
|  |  | extracellular matrix structural constituent | 5,68E-03 | 4/53 |
|  |  | extracellular structure organization | 5,68E-03 | 6/293 |
|  |  | collagen fibril organization | 1,38E-02 | 3/24 |
|  |  | **extracellular matrix** | 1,45E-02 | 5/208 |
|  |  | collagen | 1,48E-02 | 3/28 |
|  |  | proteinaceous extracellular matrix | 2,15E-02 | 4/10 |
|  |  | **collagen binding** | 2,36E-02 | 3/37 |
|  |  | extracellular matrix disassembly | 2,36E-02 | 4/120 |
|  |  | extracellular matrix part | 1,15E-01 | 3/67 |
| F6 and F7 | CPN1  CRYBB2  AKAP1  GPLD1  ACTG1 | neutral lipid catabolic process | 2,39E-02 | 3/24 |
|  |  | triglyceride catabolic process | 2,39E-02 | 3/22 |
|  |  | acylglycerol catabolic process | 2,39E-02 | 3/24 |
|  |  | glycerolipid catabolic process | 4,36E-02 | 3/32 |
|  |  | glycerolipid metabolic process | 7,98E-02 | 5/270 |
|  |  | actin filament organization | 2,25E-01 | 4/176 |
|  |  | neutral lipid metabolic process | 2,67E-01 | 3/84 |
|  |  | acylglycerol metabolic process | 2,67E-01 | 3/84 |
|  |  | glycerophospholipid metabolic process | 2,67E-01 | 4/215 |
|  |  | positive regulation of triglyceride metabolic process | 2,67E-01 | 2/14 |


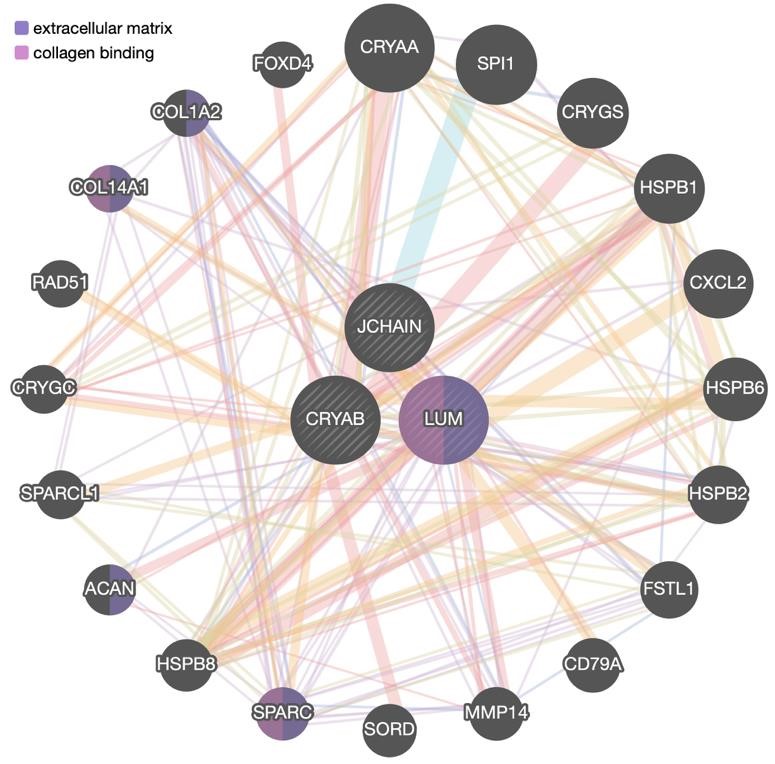


**Figure S8.** Enriched functions and gene interaction network of HC proteins only enriched on HA-coated liposomes in plasma (F4 and F5; Table S3). Extracellular matrix and collagen-binding proteins have been highlighted, along with connections between nodes to indicate physical interactions (pink), co-expression (*purple*), co-localization (*blue*) and predicted interactions (*orange*). ACAN: Aggregan LUM: Lumican; JCHAIN: Joining chain of multimeric IgA and IgM; CRYAB: Crystallin alpha B; SPARC: Secreted protein acidic and cysteine rich; COL1A2: collagen type I alpha 2 chain COL14A1 collagen type XIV alpha 1 chain.

| **Table S4.** Top 100 enriched biological functions based on the top 20 most abundant HC enriched proteins in **PLASMA**. Unknown genes excluded. Highlighted functions in network figures in bold text. | | | |
| --- | --- | --- | --- |
| **Genes** | **Function** | **FDR** | **Genes network/genome** |
| CLU  TF  FGG  IGHG1  APOA1  FGA  APOE  IGKC  C3  HP  APOA4  APOB  APOC3  IGLC2  A2M  SERPINA1  VTN  FGB  IGHA1  IGHG2 | **blood microparticle** | 2,243E-45 | 26/108 |
|  | vesicle lumen | 1,5712E-20 | 14/76 |
|  | cytoplasmic membrane-bounded vesicle lumen | 1,5712E-20 | 14/76 |
|  | **plasma lipoprotein particle** | 2,0102E-18 | 10/22 |
|  | protein-lipid complex | 2,841E-18 | 10/23 |
|  | triglyceride-rich lipoprotein particle | 5,3188E-18 | 9/15 |
|  | very-low-density lipoprotein particle | 5,3188E-18 | 9/15 |
|  | high-density lipoprotein particle | 2,2536E-17 | 9/17 |
|  | plasma lipoprotein particle remodeling | 5,4596E-16 | 9/23 |
|  | protein-lipid complex remodeling | 5,4596E-16 | 9/23 |
|  | macromolecular complex remodeling | 5,4596E-16 | 9/23 |
|  | regulation of protein processing | 5,6102E-15 | 14/200 |
|  | plasma lipoprotein particle organization | 5,6102E-15 | 9/29 |
|  | protein-lipid complex subunit organization | 7,4308E-15 | 9/30 |

| C4A  FN1  AHSG  GC  HPX  IGKV3-20  IGHV3-13  IGHV3-23  ITIH2  IGHG3  IGLC6  IGKV4-1  F2 | secretory granule lumen | 8,3976E-14 | 10/62 |
| --- | --- | --- | --- |
|  | plasma lipoprotein particle assembly | 5,1267E-13 | 7/14 |
|  | protein-lipid complex assembly | 9,0329E-13 | 7/15 |
|  | lipid localization | 9,4161E-13 | 11/118 |
|  | platelet degranulation | 1,2803E-12 | 10/82 |
|  | protein activation cascade | 6,0146E-12 | 9/61 |
|  | regulation of plasma lipoprotein particle levels | 9,1701E-12 | 8/38 |
|  | lipid transport | 9,1701E-12 | 11/147 |
|  | platelet activation | 1,2702E-11 | 12/211 |
|  | enzyme inhibitor activity | 1,4423E-11 | 12/214 |
|  | very-low-density lipoprotein particle remodeling | 2,3161E-11 | 6/11 |
|  | triglyceride-rich lipoprotein particle remodeling | 2,3161E-11 | 6/11 |
|  | steroid esterification | 4,1465E-11 | 6/12 |
|  | sterol esterification | 4,1465E-11 | 6/12 |
|  | cholesterol esterification | 4,1465E-11 | 6/12 |
|  | platelet alpha granule lumen | 4,9445E-11 | 8/48 |
|  | negative regulation of hydrolase activity | 4,9445E-11 | 12/242 |
|  | high-density lipoprotein particle remodeling | 1,2174E-10 | 6/14 |
|  | cholesterol transport | 1,439E-10 | 8/55 |
|  | sterol transport | 1,439E-10 | 8/55 |
|  | regulation of inflammatory response | 1,8369E-10 | 10/141 |
|  | reverse cholesterol transport | 2,8767E-10 | 6/16 |
|  | platelet alpha granule | 3,1686E-10 | 8/61 |
|  | secretory granule | 3,6139E-10 | 10/152 |
|  | lipoprotein particle receptor binding | 3,9949E-10 | 6/17 |
|  | positive regulation of steroid metabolic process | 3,9949E-10 | 6/17 |
|  | **complement activation** | 1,795E-09 | 7/43 |
|  | serine-type endopeptidase inhibitor activity | 2,0806E-09 | 7/44 |
|  | lipid transporter activity | 2,4027E-09 | 7/45 |
|  | exocytosis | 3,0771E-09 | 10/191 |
|  | cholesterol homeostasis | 4,9051E-09 | 7/50 |
|  | sterol homeostasis | 4,9051E-09 | 7/50 |
|  | negative regulation of endopeptidase activity | 5,4963E-09 | 9/140 |
|  | lipoprotein metabolic process | 5,4963E-09 | 8/89 |
|  | alcohol binding | 5,4963E-09 | 7/51 |
|  | negative regulation of peptidase activity | 6,1243E-09 | 9/142 |
|  | regulation of complement activation | 7,2349E-09 | 6/27 |
|  | regulation of protein activation cascade | 7,2349E-09 | 6/27 |
|  | plasma lipoprotein particle clearance | 9,0201E-09 | 6/28 |
|  | endopeptidase inhibitor activity | 1,19E-08 | 8/99 |
|  | organic hydroxy compound transport | 1,3504E-08 | 8/101 |
|  | peptidase inhibitor activity | 1,3504E-08 | 8/101 |

|  | endopeptidase regulator activity | 1,4374E-08 | 8/102 |
| --- | --- | --- | --- |
|  | negative regulation of multicellular organismal process | 1,4711E-08 | 10/230 |
|  | regulation of lipid metabolic process | 1,7025E-08 | 9/162 |
|  | cholesterol efflux | 2,324E-08 | 6/33 |
|  | regulation of humoral immune response | 2,7672E-08 | 6/34 |
|  | acute inflammatory response | 2,7672E-08 | 7/66 |
|  | phospholipid transport | 3,2335E-08 | 6/35 |
|  | lipid homeostasis | 4,0376E-08 | 7/70 |
|  | retinoid metabolic process | 4,0376E-08 | 7/70 |
|  | endocytic vesicle lumen | 4,5207E-08 | 5/16 |
|  | regulation of immune effector process | 4,6875E-08 | 9/184 |
|  | diterpenoid metabolic process | 4,7278E-08 | 7/72 |
|  | peptidase regulator activity | 7,4358E-08 | 8/128 |
|  | steroid metabolic process | 7,901E-08 | 9/197 |
|  | positive regulation of lipid metabolic process | 7,901E-08 | 7/78 |
|  | triglyceride metabolic process | 7,901E-08 | 7/78 |
|  | terpenoid metabolic process | 7,901E-08 | 7/78 |
|  | phospholipid binding | 8,5261E-08 | 9/199 |
|  | inflammatory response | 8,6772E-08 | 10/283 |
|  | acylglycerol metabolic process | 1,2598E-07 | 7/84 |
|  | neutral lipid metabolic process | 1,2598E-07 | 7/84 |
|  | regulation of steroid metabolic process | 1,4624E-07 | 6/46 |
|  | regulation of acute inflammatory response | 1,4624E-07 | 6/46 |
|  | phototransduction, visible light | 1,557E-07 | 7/87 |
|  | isoprenoid metabolic process | 1,957E-07 | 7/90 |
|  | detection of visible light | 2,6318E-07 | 7/94 |
|  | organophosphate ester transport | 2,9968E-07 | 6/52 |
|  | regulation of proteolysis | 3,1195E-07 | 8/157 |
|  | phototransduction | 3,4101E-07 | 7/98 |
|  | detection of light stimulus | 4,7894E-07 | 7/103 |
|  | regulation of endopeptidase activity | 5,6238E-07 | 9/251 |
|  | humoral immune response | 5,7299E-07 | 7/106 |
|  | regulation of sterol transport | 5,9557E-07 | 5/27 |
|  | regulation of cholesterol transport | 5,9557E-07 | 5/27 |
|  | regulation of lipoprotein lipase activity | 5,9557E-07 | 5/27 |
|  | regulation of peptidase activity | 6,7663E-07 | 9/258 |
|  | cholesterol transporter activity | 8,0095E-07 | 4/10 |
|  | glycerolipid metabolic process | 9,851E-07 | 9/270 |
|  | detection of external stimulus | 1,1954E-06 | 7/119 |
|  | sterol transporter activity | 1,2174E-06 | 4/11 |
|  | glycerolipid catabolic process | 1,3828E-06 | 5/32 |
|  | detection of abiotic stimulus | 1,4601E-06 | 7/123 |
|  | regulation of lipid catabolic process | 1,5943E-06 | 5/33 |
|  | early endosome | 1,9947E-06 | 7/129 |


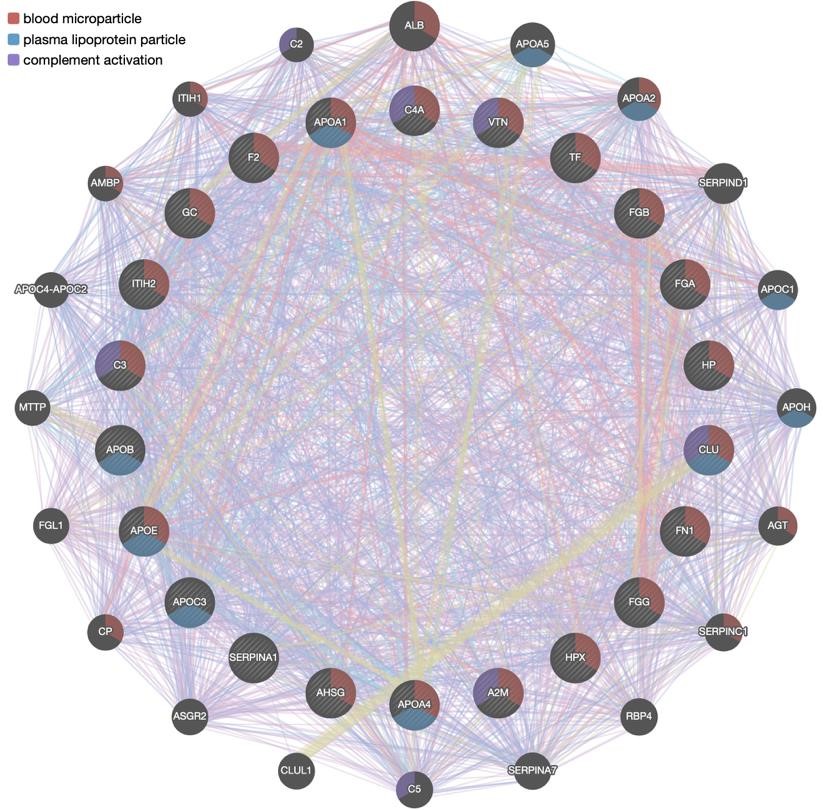


**Figure S9.** Enriched biological functions and gene interaction network based on top 20 most abundant HC proteins in **PLASMA** (Table S4). Blood microparticle, plasma lipoprotein particle and complement activation related proteins have been highlighted, along with connections between nodes to indicate physical interactions (*pink*), co-expression (*purple*), co-localization (*blue*) and predicted interactions (*orange*).

| **Table S5.** Enriched biological functions based on the top 20 most abundant HC enriched proteins in **VITREOUS**. Unknown genes and protein accession codes excluded. Highlighted functions in network figures in bold text. | | | |
| --- | --- | --- | --- |
| **Genes** | **Function** | **FDR** | **Genes network/genome** |
| GAPDH  TUBB1  TBB  ACTB  PRPH  HNRNPM  TUBA1B  PFKM  CRYBB2  LTF  HBZ  TUBB  RLBP1  ATP5F1A  CRYAA  EEF1A  CLU  RBP3  GAPDHS  TUBA1A  CRYBA1  CRYBB1  FN1  CRYAB  TUBB1  ACTG1  A2M  ALB  SNCB  SNCG  CKB  ENO1  PKM  PTGDS  DKK3  SFRP2 | **glycolysis** | 1,13E-08 | 8/48 |
|  | glucose catabolic process | 2,81E-08 | 8/58 |
|  | monosaccharide catabolic process | 1,06E-07 | 8/74 |
|  | **visual perception** | 1,06E-07 | 8/76 |
|  | hexose catabolic process | 1,06E-07 | 8/72 |
|  | sensory perception of light stimulus | 2,02E-07 | 8/84 |
|  | single-organism carbohydrate catabolic process | 5,58E-07 | 8/97 |
|  | carbohydrate catabolic process | 7,34E-07 | 8/102 |
|  | glucose metabolic process | 2,47E-05 | 8/161 |
|  | **blood microparticle** | 3,13E-05 | 7/108 |
|  | hexose metabolic process | 5,28E-05 | 8/182 |
|  | vesicle lumen | 7,86E-05 | 6/76 |
|  | 'de novo' posttranslational protein folding | 7,86E-05 | 5/38 |
|  | cytoplasmic membrane-bounded vesicle lumen | 7,86E-05 | 6/76 |
|  | monosaccharide metabolic process | 1,13E-04 | 8/209 |
|  | 'de novo' protein folding | 1,31E-04 | 5/43 |
|  | retina homeostasis | 1,74E-04 | 5/46 |
|  | secretory granule | 1,84E-04 | 7/152 |
|  | sensory perception | 2,34E-04 | 8/237 |
|  | platelet alpha granule | 6,22E-04 | 5/61 |
|  | secretory granule lumen | 6,43E-04 | 5/62 |
|  | protein folding | 9,15E-04 | 6/124 |
|  | platelet degranulation | 2,38E-03 | 5/82 |
|  | tissue homeostasis | 4,39E-03 | 5/94 |
|  | anatomical structure homeostasis | 4,39E-03 | 6/166 |
|  | platelet alpha granule lumen | 6,42E-03 | 4/48 |
|  | multicellular organismal homeostasis | 6,84E-03 | 5/105 |
|  | carbohydrate phosphorylation | 7,15E-03 | 3/16 |
|  | exocytosis | 8,15E-03 | 6/191 |
|  | carbohydrate kinase activity | 8,15E-03 | 3/17 |
|  | cytoskeleton-dependent intracellular transport | 1,32E-02 | 4/60 |
|  | cellular carbohydrate metabolic process | 3,54E-02 | 5/153 |
|  | iron ion binding | 8,09E-02 | 3/37 |


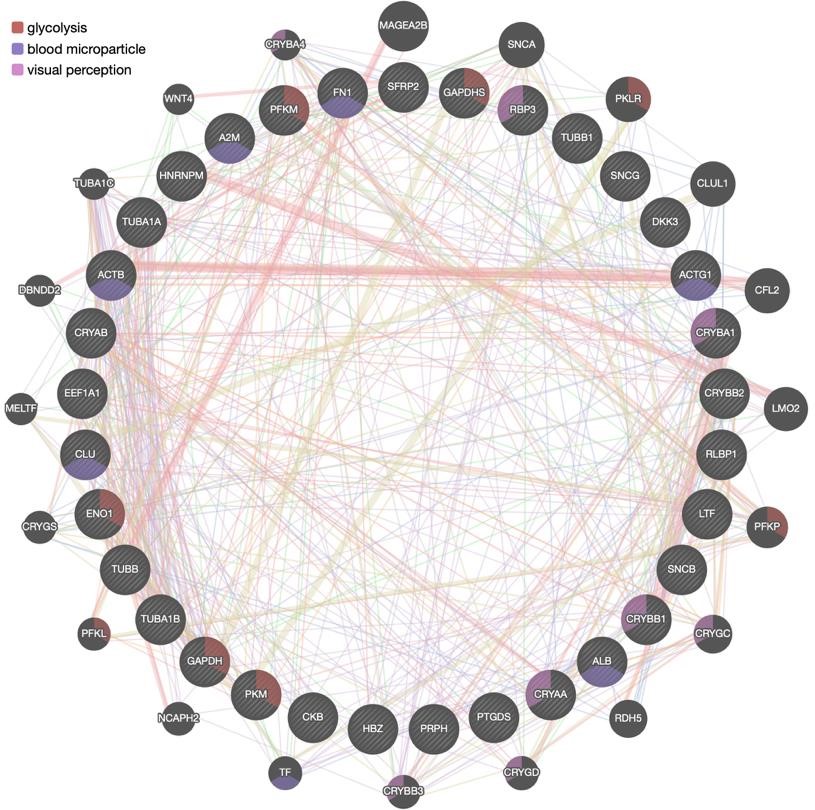


**Figure S10.** Enriched biological functions and gene interaction network based on top 20 most abundant HC proteins in **VITREOUS** (Table S5). Glycolysis, blood microparticle and visual perception related proteins have been highlighted, along with connections between nodes to indicate physical interactions (*pink*), coexpression (*purple*), co-localization (*blue*) and predicted interactions (*orange*).

**Supplementary references**

1. H.J. Yao, L. Sun, Y. Liu, S. Jiang, Y. Pu, J. Li, Y. Zhang, Monodistearoylphosphatidylethanolaminehyaluronic acid functionalization of single-walled carbon nanotubes for targeting intracellular drug delivery to overcome multidrug resistance of cancer cells, Carbon N. Y. 96 (2016) 362–376. https://doi.org/10.1016/j.carbon.2015.09.037.
2. K. Higashi, F. Mibu, K. Saito, W. Limwikrant, K. Yamamoto, K. Moribe, Composition-dependent structural changes and antitumor activity of ASC-DP/DSPE-PEG nanoparticles, Eur. J. Pharm. Sci. 99 (2017) 24–31. https://doi.org/10.1016/j.ejps.2016.11.029.
3. R. Gilli, M. Kacuráková, M. Mathlouthi, L. Navarini, S. Paoletti, FTIR studies of sodium hyaluronate and its oligomers in the amorphous solid phase and in aqueous solution, Carbohydr. Res. 263 (1994) 315–326. https://doi.org/10.1016/0008-6215(94)00147-2.
4. X. Zhao, Synthesis and characterization of a novel hyaluronic acid hydrogel, J. Biomater. Sci. Polym. Ed. 17 (2006) 419–433. https://doi.org/10.1163/156856206776374115.
5. S.L. Huang, P.X. Ling, T.M. Zhang, Oral absorption of hyaluronic acid and phospholipids complexes in rats, World J. Gastroenterol. 13 (2007) 945–949. https://doi.org/10.3748/wjg.v13.i6.945.

**Supplementary File 3**

**Top 20 PLASMA**

**PLASMA Top20 enriched**

| **Source** |  | **Protein names** | **Fold** | **GRAVY** | **pI** | **MW** | **F+Y+W** |  | **F+Y+W+H** |
| --- | --- | --- | --- | --- | --- | --- | --- | --- | --- |
| P02787 | TF | Serotransferrin | 6,54 | -0,336 | 6,81 | 77063,89 |  | 62 | 81 |
| P01876 | IGHA1 | Ig alpha-1 chain C region | 5,96 | -0,206 | 6,08 | 37654,65 |  | 23 | 31 |
| P00738 | HP | Haptoglobin | 5,96 | -0,421 | 6,13 | 45205,31 |  | 37 | 50 |
| P01834 | IGKC | Ig kappa chain C region | 5,89 | -0,537 | 6,11 | 11765,04 |  | 9 | 11 |
| P0CG05 | IGLC2 | Ig lambda-2 chain C regions | 5,41 | -0,444 | 6,91 | 11293,57 |  | 8 | 10 |
| P02675 | FGB | Fibrinogen beta chain | 5,07 | -0,758 | 8,54 | 55928,15 |  | 47 | 56 |
| P02656 | APOC3 | Apolipoprotein C-III | 4,45 | -0,086 | 5,23 | 10852,31 |  | 9 | 10 |
| P01857 | IGHG1 | Ig gamma-1 chain C region | 4,24 | -0,428 | 8,46 | 36105,91 |  | 27 | 36 |
| F5H0Q5 | AHSG | Alpha-2-HS-glycoprotein | 4,12 | -0,190 | 5,43 | 39340,74 |  | 20 | 33 |
| P02774 | GC | Vitamin D-binding protein | 3,97 | -0,336 | 5,32 | 52917,54 |  | 36 | 44 |
| P02647 | APOA1 | Apolipoprotein A-I | 3,94 | -0,717 | 5,56 | 30777,83 |  | 20 | 26 |
| P01024 | C3 | Complement C3 | 3,85 | -0,320 | 6,02 | 187148,06 |  | 134 | 163 |
| P02671 | FGA | Fibrinogen alpha chain | 3,78 | -0,822 | 5,7 | 94973,04 |  | 72 | 91 |
| P04217 | A1BG | Alpha-1B-glycoprotein | 3,40 | -0,217 | 5,56 | 54253,52 |  | 38 | 54 |
| P00734 | F2 | Prothrombin | 3,38 | -0,539 | 5,63 | 70036,87 |  | 56 | 69 |
| H0YGH4 | A2M | Alpha-2-macroglobulin | 3,18 | -0,195 | 6,03 | 163291 |  | 129 | 169 |
| P01859 | IGHG2 | Ig gamma-2 chain C region | 3,17 | -0,419 | 7,66 | 35900,64 |  | 28 | 36 |
| P02679-2 | FGG | Fibrinogen gamma chain | 3,10 | -0,588 | 5,37 | 51511,66 |  | 55 | 67 |
| P01860 | IGHG3 | Ig gamma-3 chain C region | 2,90 | -0,511 | 8,23 | 41286,96 |  | 27 | 35 |
| P08603 | CFH | Complement factor H | 2,87 | -0,603 | 6,21 | 139096,31 |  | 126 | 154 |
|  | **Average** |  | **4,26** | **-0,434** | **6,35** | **62320,15** |  | **48,15** | **61,30** |
|  | **SD** |  | **1,15** | **0,203** | **1,07** | **48874,87** |  | **39,34** | **48,74** |
| **F1** | **HC** |  |  |  |  |  |  |  |  |
| P10909-4 | CLU | Clusterin | 7,32 | -0,665 | 5,88 | 52494,58 |  | 39 | 52 |
| P02787 | TF | Serotransferrin | 6,79 | -0,336 | 6,81 | 77063,89 |  | 62 | 81 |
| P02679-2 | FGG | Fibrinogen gamma chain | 6,45 | -0,588 | 5,37 | 51511,66 |  | 55 | 67 |
| P01857 | IGHG1 | Ig gamma-1 chain C region | 6,31 | -0,428 | 8,46 | 36105,91 |  | 27 | 36 |
| P02647 | APOA1 | Apolipoprotein A-I | 5,98 | -0,717 | 5,56 | 30777,83 |  | 20 | 26 |
| P02671 | FGA | Fibrinogen alpha chain | 5,74 | -0,822 | 5,7 | 94973,04 |  | 72 | 91 |
| P02649 | APOE | Apolipoprotein E | 5,73 | -0,596 | 5,65 | 36154,08 |  | 16 | 18 |
| P01834 | IGKC | Ig kappa chain C region | 5,68 | -0,537 | 6,11 | 11765,04 |  | 9 | 11 |
| P01024 | C3 | Complement C3 | 5,60 | -0,320 | 6,02 | 187148,06 |  | 134 | 163 |
| P00738 | HP | Haptoglobin | 5,57 | -0,421 | 6,13 | 45205,31 |  | 37 | 50 |
| P06727 | APOA4 | Apolipoprotein A-IV | 5,41 | -0,796 | 5,28 | 45399,06 |  | 20 | 28 |
| P04114 | APOB | Apolipoprotein B-100 | 5,37 | -0,296 | 6,58 | 515604,73 |  | 414 | 528 |
| P02656 | APOC3 | Apolipoprotein C-III | 5,30 | -0,086 | 5,23 | 10852,31 |  | 9 | 10 |
| P0CG05 | IGLC2 | Ig lambda-2 chain C regions | 5,01 | -0,444 | 6,91 | 11293,57 |  | 8 | 10 |
| H0YGH4 | A2M | Alpha-2-macroglobulin | 4,95 | -0,195 | 6,03 | 163291 |  | 129 | 169 |
| P01009 | SERPINA1 | Alpha-1-antitrypsin | 4,49 | -0,183 | 5,37 | 46736,55 |  | 36 | 49 |
| P04004 | VTN | Vitronectin | 4,29 | -0,723 | 5,55 | 54305,59 |  | 56 | 65 |
| P02675 | FGB | Fibrinogen beta chain | 4,28 | -0,758 | 8,54 | 55928,15 |  | 47 | 56 |
| P01876 | IGHA1 | Ig alpha-1 chain C region | 4,03 | -0,206 | 6,08 | 37654,65 |  | 23 | 31 |
| P01859 | IGHG2 | Ig gamma-2 chain C region | 4,00 | -0,419 | 7,66 | 35900,64 |  | 28 | 36 |
|  | **Average** |  | **5,42** | **-0,477** | **6,25** | **80008,28** |  | **62,05** | **78,85** |
|  | **SD** |  | **0,91** | **0,225** | **0,98** | **112130,65** |  | **90,00** | **114,60** |
| **F1** | **SC** |  |  |  |  |  |  |  |  |
| P02787 | TF | Serotransferrin | 6,29 | -0,336 | 6,81 | 77063,89 |  | 62 | 81 |
| P01857 | IGHG1 | Ig gamma-1 chain C region | 5,91 | -0,428 | 8,46 | 36105,91 |  | 27 | 36 |
| P02647 | APOA1 | Apolipoprotein A-I | 5,57 | -0,717 | 5,56 | 30777,83 |  | 20 | 26 |
| P01834 | IGKC | Ig kappa chain C region | 5,41 | -0,537 | 6,11 | 11765,04 |  | 9 | 11 |
| H0YGH4 | A2M | Alpha-2-macroglobulin | 5,04 | -0,195 | 6,03 | 163291 |  | 129 | 169 |
| P00738 | HP | Haptoglobin | 5,02 | -0,421 | 6,13 | 45205,31 |  | 37 | 50 |
| P01876 | IGHA1 | Ig alpha-1 chain C region | 4,59 | -0,206 | 6,08 | 37654,65 |  | 23 | 31 |
| P01024 | C3 | Complement C3 | 4,59 | -0,320 | 6,02 | 187148,06 |  | 134 | 163 |
| P02679-2 | FGG | Fibrinogen gamma chain | 4,50 | -0,588 | 5,37 | 51511,66 |  | 55 | 67 |
| P0CG05 | IGLC2 | Ig lambda-2 chain C regions | 4,50 | -0,444 | 6,91 | 11293,57 |  | 8 | 10 |
| P02675 | FGB | Fibrinogen beta chain | 4,45 | -0,758 | 8,54 | 55928,15 |  | 47 | 56 |
| P01009 | SERPINA1 | Alpha-1-antitrypsin | 4,12 | -0,183 | 5,37 | 46736,55 |  | 36 | 49 |
| P02671 | FGA | Fibrinogen alpha chain | 3,87 | -0,822 | 5,7 | 94973,04 |  | 72 | 91 |
| P02790 | HPX | Hemopexin | 3,39 | -0,434 | 6,55 | 51676,37 |  | 55 | 74 |
| P01859 | IGHG2 | Ig gamma-2 chain C region | 3,32 | -0,419 | 7,66 | 35900,64 |  | 28 | 36 |
| B0V2C8 | C4A | Complement C4-A | 2,80 | -0,252 | 6,66 | 192785,48 |  | 139 | 177 |
| P01860 | IGHG3 | Ig gamma-3 chain C region | 2,66 | -0,511 | 8,23 | 41286,96 |  | 27 | 35 |
| P01871 | IGHM | Ig mu chain C region | 2,36 | -0,319 | 6,35 | 49439,74 |  | 35 | 44 |
| P06727 | APOA4 | Apolipoprotein A-IV | 2,34 | -0,796 | 5,28 | 45399,06 |  | 20 | 28 |
| P02652 | APOA2 | Apolipoprotein A-II | 2,25 | -0,084 | 6,27 | 11175,02 |  | 8 | 8 |
|  | **Average** |  | **4,15** | **-0,439** | **6,50** | **63855,90** |  | **48,55** | **62,10** |
|  | **SD** |  | **1,24** | **0,214** | **1,01** | **54531,47** |  | **40,87** | **51,62** |
| **F2** | **HC** |  |  |  |  |  |  |  |  |
| P02787 | TF | Serotransferrin | 6,78 | -0,336 | 6,81 | 77063,89 |  | 62 | 81 |
| P10909-4 | CLU | Clusterin | 6,59 | -0,665 | 5,88 | 52494,58 |  | 39 | 52 |

| P01857 | IGHG1 | Ig gamma-1 chain C region | 6,46 | -0,428 | 8,46 | 36105,91 | 27 | 36 |
| --- | --- | --- | --- | --- | --- | --- | --- | --- |
| P01834 | IGKC | Ig kappa chain C region | 5,86 | -0,537 | 6,11 | 11765,04 | 9 | 11 |
| H0YGH4 | A2M | Alpha-2-macroglobulin | 5,69 | -0,195 | 6,03 | 163291 | 129 | 169 |
| P02671 | FGA | Fibrinogen alpha chain | 5,64 | -0,822 | 5,7 | 94973,04 | 72 | 91 |
| P01024 | C3 | Complement C3 | 5,55 | -0,320 | 6,02 | 187148,06 | 134 | 163 |
| P0CG05 | IGLC2 | Ig lambda-2 chain C regions | 5,53 | -0,444 | 6,91 | 11293,57 | 8 | 10 |
| P02675 | FGB | Fibrinogen beta chain | 5,31 | -0,758 | 8,54 | 55928,15 | 47 | 56 |
| P01876 | IGHA1 | Ig alpha-1 chain C region | 5,24 | -0,206 | 6,08 | 37654,65 | 23 | 31 |
| P02649 | APOE | Apolipoprotein E | 5,23 | -0,596 | 5,65 | 36154,08 | 16 | 18 |
| P02679-2 | FGG | Fibrinogen gamma chain | 4,91 | -0,588 | 5,37 | 51511,66 | 55 | 67 |
| P02647 | APOA1 | Apolipoprotein A-I | 4,88 | -0,717 | 5,56 | 30777,83 | 20 | 26 |
| P01009 | SERPINA1 | Alpha-1-antitrypsin | 4,83 | -0,183 | 5,37 | 46736,55 | 36 | 49 |
| P02656 | APOC3 | Apolipoprotein C-III | 4,69 | -0,086 | 5,23 | 10852,31 | 9 | 10 |
| P04114 | APOB | Apolipoprotein B-100 | 4,45 | -0,296 | 6,58 | 515604,73 | 414 | 528 |
| B0V2C8 | C4A | Complement C4-A | 4,43 | -0,252 | 6,66 | 192785,48 | 139 | 177 |
| P02751-14 | FN1 | Fibronectin | 4,11 | -0,514 | 5,31 | 272320,36 | 198 | 249 |
| P00738 | HP | Haptoglobin | 3,93 | -0,421 | 6,13 | 45205,31 | 37 | 50 |
| P01859 | IGHG2 | Ig gamma-2 chain C region | 3,85 | -0,419 | 7,66 | 35900,64 | 28 | 36 |
|  | **Average** |  | **5,20** | **-0,439** | **6,30** | **98278,34** | **75,10** | **95,50** |
|  | **SD** |  | **0,84** | **0,208** | **0,97** | **121408,55** | **95,45** | **121,35** |
| **F2** | **SC** |  |  |  |  |  |  |  |
| P02787 | TF | Serotransferrin | 6,55 | -0,336 | 6,81 | 77063,89 | 62 | 81 |
| P01857 | IGHG1 | Ig gamma-1 chain C region | 6,39 | -0,428 | 8,46 | 36105,91 | 27 | 36 |
| P02647 | APOA1 | Apolipoprotein A-I | 5,95 | -0,717 | 5,56 | 30777,83 | 20 | 26 |
| P01834 | IGKC | Ig kappa chain C region | 5,83 | -0,537 | 6,11 | 11765,04 | 9 | 11 |
| P00738 | HP | Haptoglobin | 5,30 | -0,421 | 6,13 | 45205,31 | 37 | 50 |
| H0YGH4 | A2M | Alpha-2-macroglobulin | 5,09 | -0,195 | 6,03 | 163291 | 129 | 169 |
| P01024 | C3 | Complement C3 | 5,01 | -0,320 | 6,02 | 187148,06 | 134 | 163 |
| P01876 | IGHA1 | Ig alpha-1 chain C region | 4,82 | -0,206 | 6,08 | 37654,65 | 23 | 31 |
| P02679-2 | FGG | Fibrinogen gamma chain | 4,81 | -0,588 | 5,37 | 51511,66 | 55 | 67 |
| P0CG05 | IGLC2 | Ig lambda-2 chain C regions | 4,61 | -0,444 | 6,91 | 11293,57 | 8 | 10 |
| P02675 | FGB | Fibrinogen beta chain | 4,60 | -0,758 | 8,54 | 55928,15 | 47 | 56 |
| P02671 | FGA | Fibrinogen alpha chain | 4,28 | -0,822 | 5,7 | 94973,04 | 72 | 91 |
| P02790 | HPX | Hemopexin | 3,66 | -0,434 | 6,55 | 51676,37 | 55 | 74 |
| P01859 | IGHG2 | Ig gamma-2 chain C region | 3,56 | -0,419 | 7,66 | 35900,64 | 28 | 36 |
| P01009 | SERPINA1 | Alpha-1-antitrypsin | 3,26 | -0,183 | 5,37 | 46736,55 | 36 | 49 |
| B0V2C8 | C4A | Complement C4-A | 3,07 | -0,252 | 6,66 | 192785,48 | 139 | 177 |
| P02652 | APOA2 | Apolipoprotein A-II | 3,07 | -0,084 | 6,27 | 11175,02 | 8 | 8 |
| P01860 | IGHG3 | Ig gamma-3 chain C region | 2,93 | -0,511 | 8,23 | 41286,96 | 27 | 35 |
| P06727 | APOA4 | Apolipoprotein A-IV | 2,89 | -0,796 | 5,28 | 45399,06 | 20 | 28 |
| P02749 | APOH | Beta-2-glycoprotein 1 | 2,62 | -0,274 | 8,34 | 38298,16 | 39 | 45 |
|  | **Average** |  | **4,41** | **-0,436** | **6,60** | **63298,82** | **48,75** | **62,15** |
|  | **SD** |  | **1,23** | **0,216** | **1,08** | **54742,99** | **40,81** | **51,61** |
| **F3** | **HC** |  |  |  |  |  |  |  |
| P02787 | TF | Serotransferrin | 7,13 | -0,336 | 6,81 | 77063,89 | 62 | 81 |
| P01857 | IGHG1 | Ig gamma-1 chain C region | 6,43 | -0,428 | 8,46 | 36105,91 | 27 | 36 |
| P01834 | IGKC | Ig kappa chain C region | 6,36 | -0,537 | 6,11 | 11765,04 | 9 | 11 |
| H0YGH4 | A2M | Alpha-2-macroglobulin | 5,96 | -0,195 | 6,03 | 163291 | 129 | 169 |
| P00738 | HP | Haptoglobin | 5,92 | -0,421 | 6,13 | 45205,31 | 37 | 50 |
| P0CG05 | IGLC2 | Ig lambda-2 chain C regions | 5,92 | -0,444 | 6,91 | 11293,57 | 8 | 10 |
| P01024 | C3 | Complement C3 | 5,76 | -0,320 | 6,02 | 187148,06 | 134 | 163 |
| P02679-2 | FGG | Fibrinogen gamma chain | 5,72 | -0,588 | 5,37 | 51511,66 | 55 | 67 |
| P10909-4 | CLU | Clusterin | 5,64 | -0,665 | 5,88 | 52494,58 | 39 | 52 |
| P01876 | IGHA1 | Ig alpha-1 chain C region | 5,52 | -0,206 | 6,08 | 37654,65 | 23 | 31 |
| P02671 | FGA | Fibrinogen alpha chain | 5,49 | -0,822 | 5,7 | 94973,04 | 72 | 91 |
| P02675 | FGB | Fibrinogen beta chain | 5,40 | -0,758 | 8,54 | 55928,15 | 47 | 56 |
| P02647 | APOA1 | Apolipoprotein A-I | 4,99 | -0,717 | 5,56 | 30777,83 | 20 | 26 |
| P06727 | APOA4 | Apolipoprotein A-IV | 4,97 | -0,796 | 5,28 | 45399,06 | 20 | 28 |
| F5H0Q5 | AHSG | Alpha-2-HS-glycoprotein | 4,45 | -0,190 | 5,43 | 39340,74 | 20 | 33 |
| P01859 | IGHG2 | Ig gamma-2 chain C region | 4,18 | -0,419 | 7,66 | 35900,64 | 28 | 36 |
| P04114 | APOB | Apolipoprotein B-100 | 4,15 | -0,296 | 6,58 | 515604,73 | 414 | 528 |
| P02774 | GC | Vitamin D-binding protein | 4,06 | -0,336 | 5,32 | 52917,54 | 36 | 44 |
| P02790 | HPX | Hemopexin | 3,94 | -0,434 | 6,55 | 51676,37 | 55 | 74 |
| P02656 | APOC3 | Apolipoprotein C-III | 3,60 | -0,086 | 5,23 | 10852,31 | 9 | 10 |
|  | **Average** |  | **5,28** | **-0,450** | **6,28** | **80345,20** | **62,20** | **79,80** |
|  | **SD** |  | **0,95** | **0,217** | **0,98** | **112012,63** | **89,90** | **114,26** |
| **F3** | **SC** |  |  |  |  |  |  |  |
| P02787 | TF | Serotransferrin | 5,87 | -0,336 | 6,81 | 77063,89 | 62 | 81 |
| P01857 | IGHG1 | Ig gamma-1 chain C region | 5,58 | -0,428 | 8,46 | 36105,91 | 27 | 36 |
| P02647 | APOA1 | Apolipoprotein A-I | 5,20 | -0,717 | 5,56 | 30777,83 | 20 | 26 |
| P01834 | IGKC | Ig kappa chain C region | 4,96 | -0,537 | 6,11 | 11765,04 | 9 | 11 |
| P00738 | HP | Haptoglobin | 4,70 | -0,421 | 6,13 | 45205,31 | 37 | 50 |
| H0YGH4 | A2M | Alpha-2-macroglobulin | 4,60 | -0,195 | 6,03 | 163291 | 129 | 169 |

| P01876 | IGHA1 | Ig alpha-1 chain C region | 4,20 | -0,206 | 6,08 | 37654,65 | 23 | 31 |
| --- | --- | --- | --- | --- | --- | --- | --- | --- |
| P01024 | C3 | Complement C3 | 4,07 | -0,320 | 6,02 | 187148,06 | 134 | 163 |
| P0CG05 | IGLC2 | Ig lambda-2 chain C regions | 4,02 | -0,444 | 6,91 | 11293,57 | 8 | 10 |
| P01859 | IGHG2 | Ig gamma-2 chain C region | 3,90 | -0,419 | 7,66 | 35900,64 | 28 | 36 |
| P02671 | FGA | Fibrinogen alpha chain | 3,66 | -0,822 | 5,7 | 94973,04 | 72 | 91 |
| P02679-2 | FGG | Fibrinogen gamma chain | 3,58 | -0,588 | 5,37 | 51511,66 | 55 | 67 |
| P06727 | APOA4 | Apolipoprotein A-IV | 3,44 | -0,796 | 5,28 | 45399,06 | 20 | 28 |
| P01009 | SERPINA1 | Alpha-1-antitrypsin | 3,30 | -0,183 | 5,37 | 46736,55 | 36 | 49 |
| P02675 | FGB | Fibrinogen beta chain | 2,87 | -0,758 | 8,54 | 55928,15 | 47 | 56 |
| P02790 | HPX | Hemopexin | 2,86 | -0,434 | 6,55 | 51676,37 | 55 | 74 |
| P01860 | IGHG3 | Ig gamma-3 chain C region | 2,06 | -0,511 | 8,23 | 41286,96 | 27 | 35 |
|  | **Average** |  | **4,05** | **-0,477** | **6,52** | **60218,69** | **46,41** | **59,59** |
|  | **SD** |  | **1,02** | **0,205** | **1,09** | **47872,95** | **36,78** | **46,15** |
| **F4** | **HC** |  |  |  |  |  |  |  |
| P01024 | C3 | Complement C3 | 4,61 | -0,320 | 6,02 | 187148,06 | 134 | 163 |
| P01857 | IGHG1 | Ig gamma-1 chain C region | 4,35 | -0,428 | 8,46 | 36105,91 | 27 | 36 |
| P02675 | FGB | Fibrinogen beta chain | 4,12 | -0,758 | 8,54 | 55928,15 | 47 | 56 |
| P00738 | HP | Haptoglobin | 3,44 | -0,421 | 6,13 | 45205,31 | 37 | 50 |
| P02787 | TF | Serotransferrin | 3,36 | -0,336 | 6,81 | 77063,89 | 62 | 81 |
| P01834 | IGKC | Ig kappa chain C region | 3,15 | -0,537 | 6,11 | 11765,04 | 9 | 11 |
| P01619 | IGKV3-20 | Ig kappa chain V-III region WOL | 2,97 | -0,130 | 4,85 | 12557,12 | 12 | 12 |
| H0YGH4 | A2M | Alpha-2-macroglobulin | 2,97 | -0,195 | 6,03 | 163291 | 129 | 169 |
| P01766 | IGHV3-13 | Ig heavy chain V-III region BRO | 2,87 | 0,066 | 6,54 | 12506,22 | 13 | 14 |
| P02671 | FGA | Fibrinogen alpha chain | 2,77 | -0,822 | 5,7 | 94973,04 | 72 | 91 |
| P02751-14 | FN1 | Fibronectin | 2,03 | -0,514 | 5,31 | 272320,36 | 198 | 249 |
| P02679-2 | FGG | Fibrinogen gamma chain | 1,66 | -0,588 | 5,37 | 51511,66 | 55 | 67 |
| P04004 | VTN | Vitronectin | 1,34 | -0,723 | 5,55 | 54305,59 | 56 | 65 |
| P01764 | IGHV3-23 | Ig heavy chain V-III region TIL | 1,13 | 0,073 | 8,49 | 12582,31 | 14 | 14 |
| F5H0Q5 | AHSG | Alpha-2-HS-glycoprotein | 1,09 | -0,190 | 5,43 | 39340,74 | 20 | 33 |
| Q5T985 | ITIH2 | Inter-alpha-trypsin inhibitor heavy chain H2 | 1,05 | -0,293 | 6,4 | 106463,47 | 87 | 110 |
| P04114 | APOB | Apolipoprotein B-100 | 1,02 | -0,296 | 6,58 | 515604,73 | 414 | 528 |
| P01860 | IGHG3 | Ig gamma-3 chain C region | 0,86 | -0,511 | 8,23 | 41286,96 | 27 | 35 |
| P01859 | IGHG2 | Ig gamma-2 chain C region | 0,76 | -0,419 | 7,66 | 35900,64 | 28 | 36 |
| P0CF74 | IGLC6 | Ig lambda-6 chain C region | 0,64 | -0,467 | 6,91 | 11276,54 | 8 | 10 |
|  | **Average** |  | **2,31** | **-0,391** | **6,56** | **91856,84** | **72,45** | **91,50** |
|  | **SD** |  | **1,30** | **0,244** | **1,16** | **120654,22** | **94,55** | **120,40** |
| **F4** | **SC** |  |  |  |  |  |  |  |
| H0YGH4 | A2M | Alpha-2-macroglobulin | 6,44 | -0,195 | 6,03 | 163291 | 129 | 169 |
| P02787 | TF | Serotransferrin | 6,35 | -0,336 | 6,81 | 77063,89 | 62 | 81 |
| P01857 | IGHG1 | Ig gamma-1 chain C region | 6,08 | -0,428 | 8,46 | 36105,91 | 27 | 36 |
| P01009 | SERPINA1 | Alpha-1-antitrypsin | 5,71 | -0,183 | 5,37 | 46736,55 | 36 | 49 |
| P01024 | C3 | Complement C3 | 5,46 | -0,320 | 6,02 | 187148,06 | 134 | 163 |
| P00738 | HP | Haptoglobin | 5,23 | -0,421 | 6,13 | 45205,31 | 37 | 50 |
| P02679-2 | FGG | Fibrinogen gamma chain | 5,21 | -0,588 | 5,37 | 51511,66 | 55 | 67 |
| P01876 | IGHA1 | Ig alpha-1 chain C region | 5,17 | -0,206 | 6,08 | 37654,65 | 23 | 31 |
| P02675 | FGB | Fibrinogen beta chain | 5,10 | -0,758 | 8,54 | 55928,15 | 47 | 56 |
| P01834 | IGKC | Ig kappa chain C region | 5,03 | -0,537 | 6,11 | 11765,04 | 9 | 11 |
| P01860 | IGHG3 | Ig gamma-3 chain C region | 4,98 | -0,511 | 8,23 | 41286,96 | 27 | 35 |
| P02671 | FGA | Fibrinogen alpha chain | 4,79 | -0,822 | 5,7 | 94973,04 | 72 | 91 |
| P02647 | APOA1 | Apolipoprotein A-I | 4,50 | -0,717 | 5,56 | 30777,83 | 20 | 26 |
| P02790 | HPX | Hemopexin | 4,03 | -0,434 | 6,55 | 51676,37 | 55 | 74 |
| P0CF74 | IGLC6 | Ig lambda-6 chain C region | 3,75 | -0,467 | 6,91 | 11276,54 | 8 | 10 |
| P0CG05 | IGLC2 | Ig lambda-2 chain C regions | 3,75 | -0,444 | 6,91 | 11293,57 | 8 | 10 |
| P0CG06 | IGLC3 | Ig lambda-3 chain C regions | 3,75 | -0,439 | 6,91 | 11265,56 | 8 | 10 |
| P01859 | IGHG2 | Ig gamma-2 chain C region | 3,73 | -0,419 | 7,66 | 35900,64 | 28 | 36 |
| P02763 | ORM1 | Alpha-1-acid glycoprotein 1 | 3,43 | -0,536 | 4,93 | 23511,56 | 24 | 27 |
| P00450 | CP | Ceruloplasmin | 3,39 | -0,536 | 5,44 | 122205,19 | 135 | 176 |
|  | **Average** |  | **4,79** | **-0,465** | **6,49** | **57328,87** | **47,20** | **60,40** |
|  | **SD** |  | **0,96** | **0,174** | **1,06** | **49345,16** | **41,19** | **52,61** |
| **F5** | **HC** |  |  |  |  |  |  |  |
| P01857 | IGHG1 | Ig gamma-1 chain C region | 4,69 | -0,428 | 8,46 | 36105,91 | 27 | 36 |
| P02671 | FGA | Fibrinogen alpha chain | 4,66 | -0,822 | 5,7 | 94973,04 | 72 | 91 |
| P01024 | C3 | Complement C3 | 4,61 | -0,320 | 6,02 | 187148,06 | 134 | 163 |
| P01619 | IGKV3-20 | Ig kappa chain V-III region WOL | 4,25 | -0,130 | 4,85 | 12557,12 | 12 | 12 |
| P02675 | FGB | Fibrinogen beta chain | 4,11 | -0,758 | 8,54 | 55928,15 | 47 | 56 |
| P00738 | HP | Haptoglobin | 3,96 | -0,421 | 6,13 | 45205,31 | 37 | 50 |
| P02787 | TF | Serotransferrin | 3,71 | -0,336 | 6,81 | 77063,89 | 62 | 81 |
| P01834 | IGKC | Ig kappa chain C region | 3,20 | -0,537 | 6,11 | 11765,04 | 9 | 11 |
| H0YGH4 | A2M | Alpha-2-macroglobulin | 3,14 | -0,195 | 6,03 | 163291 | 129 | 169 |
| P06312 | IGKV4-1 | Ig kappa chain V-IV region Len | 2,74 | -0,143 | 5,09 | 13380,05 | 15 | 15 |
| P01766 | IGHV3-13 | Ig heavy chain V-III region BRO | 2,17 | 0,066 | 6,54 | 12506,22 | 13 | 14 |
| P0CG05 | IGLC2 | Ig lambda-2 chain C regions | 2,05 | -0,444 | 6,91 | 11293,57 | 8 | 10 |
| B0V2C8 | C4A | Complement C4-A | 1,93 | -0,252 | 6,66 | 192785,48 | 139 | 177 |

| P02751-14 | FN1 | Fibronectin | 1,88 | -0,514 | 5,31 | 272320,36 | 198 | 249 |
| --- | --- | --- | --- | --- | --- | --- | --- | --- |
| F5H0Q5 | AHSG | Alpha-2-HS-glycoprotein | 1,71 | -0,190 | 5,43 | 39340,74 | 20 | 33 |
| P01859 | IGHG2 | Ig gamma-2 chain C region | 1,41 | -0,419 | 7,66 | 35900,64 | 28 | 36 |
| P04114 | APOB | Apolipoprotein B-100 | 1,32 | -0,296 | 6,58 | 515604,73 | 414 | 528 |
| P02679-2 | FGG | Fibrinogen gamma chain | 1,24 | -0,588 | 5,37 | 51511,66 | 55 | 67 |
| Q5T985 | ITIH2 | Inter-alpha-trypsin inhibitor heavy chain H2 | 1,12 | -0,293 | 6,4 | 106463,47 | 87 | 110 |
| P01876 | IGHA1 | Ig alpha-1 chain C region | 1,10 | -0,206 | 6,08 | 37654,65 | 23 | 31 |
|  | **Average** |  | **2,75** | **-0,361** | **6,33** | **98639,95** | **76,45** | **96,95** |
|  | **SD** |  | **1,31** | **0,215** | **1,01** | **122409,93** | **95,68** | **121,77** |
| **F5** | **SC** |  |  |  |  |  |  |  |
| P01857 | IGHG1 | Ig gamma-1 chain C region | 6,32 | -0,428 | 8,46 | 36105,91 | 27 | 36 |
| P02647 | APOA1 | Apolipoprotein A-I | 6,23 | -0,717 | 5,56 | 30777,83 | 20 | 26 |
| P02787 | TF | Serotransferrin | 6,04 | -0,336 | 6,81 | 77063,89 | 62 | 81 |
| P02671 | FGA | Fibrinogen alpha chain | 5,66 | -0,822 | 5,7 | 94973,04 | 72 | 91 |
| H0YGH4 | A2M | Alpha-2-macroglobulin | 5,55 | -0,195 | 6,03 | 163291 | 129 | 169 |
| P01024 | C3 | Complement C3 | 5,54 | -0,320 | 6,02 | 187148,06 | 134 | 163 |
| P00738 | HP | Haptoglobin | 5,53 | -0,421 | 6,13 | 45205,31 | 37 | 50 |
| P01009 | SERPINA1 | Alpha-1-antitrypsin | 5,49 | -0,183 | 5,37 | 46736,55 | 36 | 49 |
| P02675 | FGB | Fibrinogen beta chain | 5,31 | -0,758 | 8,54 | 55928,15 | 47 | 56 |
| P01876 | IGHA1 | Ig alpha-1 chain C region | 5,30 | -0,206 | 6,08 | 37654,65 | 23 | 31 |
| Q5T985 | ITIH2 | Inter-alpha-trypsin inhibitor heavy chain H2 | 5,30 | -0,293 | 6,4 | 106463,47 | 87 | 110 |
| P0CG05 | IGLC2 | Ig lambda-2 chain C regions | 4,91 | -0,444 | 6,91 | 11293,57 | 8 | 10 |
| P01834 | IGKC | Ig kappa chain C region | 4,53 | -0,537 | 6,11 | 11765,04 | 9 | 11 |
| Q9BUG6 | ZSCAN5A | Zinc finger and SCAN domain-containing protein 5A | 4,39 | -0,827 | 8,7 | 55865,34 | 22 | 42 |
| P02790 | HPX | Hemopexin | 4,05 | -0,434 | 6,55 | 51676,37 | 55 | 74 |
| P02679-2 | FGG | Fibrinogen gamma chain | 4,01 | -0,588 | 5,37 | 51511,66 | 55 | 67 |
| F5H0Q5 | AHSG | Alpha-2-HS-glycoprotein | 3,83 | -0,190 | 5,43 | 39340,74 | 20 | 33 |
| P02763 | ORM1 | Alpha-1-acid glycoprotein 1 | 3,71 | -0,536 | 4,93 | 23511,56 | 24 | 27 |
| P01859 | IGHG2 | Ig gamma-2 chain C region | 3,71 | -0,419 | 7,66 | 35900,64 | 28 | 36 |
| P06727 | APOA4 | Apolipoprotein A-IV | 3,57 | -0,796 | 5,28 | 45399,06 | 20 | 28 |
|  | **Average** |  | **4,95** | **-0,473** | **6,40** | **60380,59** | **45,75** | **59,50** |
|  | **SD** |  | **0,90** | **0,219** | **1,13** | **46026,88** | **36,06** | **44,77** |
| **F6** | **HC** |  |  |  |  |  |  |  |
| P02671 | FGA | Fibrinogen alpha chain | 5,56 | -0,822 | 5,7 | 94973,04 | 72 | 91 |
| P02675 | FGB | Fibrinogen beta chain | 4,95 | -0,758 | 8,54 | 55928,15 | 47 | 56 |
| P01619 | IGKV3-20 | Ig kappa chain V-III region WOL | 4,75 | -0,130 | 4,85 | 12557,12 | 12 | 12 |
| P02787 | TF | Serotransferrin | 4,70 | -0,336 | 6,81 | 77063,89 | 62 | 81 |
| P01857 | IGHG1 | Ig gamma-1 chain C region | 4,43 | -0,428 | 8,46 | 36105,91 | 27 | 36 |
| P00738 | HP | Haptoglobin | 4,30 | -0,421 | 6,13 | 45205,31 | 37 | 50 |
| P01024 | C3 | Complement C3 | 3,91 | -0,320 | 6,02 | 187148,06 | 134 | 163 |
| P01834 | IGKC | Ig kappa chain C region | 3,56 | -0,537 | 6,11 | 11765,04 | 9 | 11 |
| F5H0Q5 | AHSG | Alpha-2-HS-glycoprotein | 3,24 | -0,190 | 5,43 | 39340,74 | 20 | 33 |
| H0YGH4 | A2M | Alpha-2-macroglobulin | 2,90 | -0,195 | 6,03 | 163291 | 129 | 169 |
| P00734 | F2 | Prothrombin | 2,57 | -0,539 | 5,63 | 70036,87 | 56 | 69 |
| P10909-4 | CLU | Clusterin | 2,38 | -0,665 | 5,88 | 52494,58 | 39 | 52 |
| P01876 | IGHA1 | Ig alpha-1 chain C region | 2,37 | -0,206 | 6,08 | 37654,65 | 23 | 31 |
| P01766 | IGHV3-13 | Ig heavy chain V-III region BRO | 2,09 | 0,066 | 6,54 | 12506,22 | 13 | 14 |
| P04004 | VTN | Vitronectin | 2,01 | -0,723 | 5,55 | 54305,59 | 56 | 65 |
| P0CG05 | IGLC2 | Ig lambda-2 chain C regions | 1,93 | -0,444 | 6,91 | 11293,57 | 8 | 10 |
|  | **Average** |  | **3,48** | **-0,416** | **6,29** | **60104,36** | **46,50** | **58,94** |
|  | **SD** |  | **1,20** | **0,251** | **1,00** | **51301,20** | **38,74** | **48,74** |
| **F6** | **SC** |  |  |  |  |  |  |  |
| P02675 | FGB | Fibrinogen beta chain | 6,03 | -0,758 | 8,54 | 55928,15 | 47 | 56 |
| P01857 | IGHG1 | Ig gamma-1 chain C region | 5,84 | -0,428 | 8,46 | 36105,91 | 27 | 36 |
| P02787 | TF | Serotransferrin | 5,77 | -0,336 | 6,81 | 77063,89 | 62 | 81 |
| P02671 | FGA | Fibrinogen alpha chain | 5,63 | -0,822 | 5,7 | 94973,04 | 72 | 91 |
| P01009 | SERPINA1 | Alpha-1-antitrypsin | 5,63 | -0,183 | 5,37 | 46736,55 | 36 | 49 |
| P02647 | APOA1 | Apolipoprotein A-I | 5,58 | -0,717 | 5,56 | 30777,83 | 20 | 26 |
| P00738 | HP | Haptoglobin | 5,42 | -0,421 | 6,13 | 45205,31 | 37 | 50 |
| P01876 | IGHA1 | Ig alpha-1 chain C region | 5,28 | -0,206 | 6,08 | 37654,65 | 23 | 31 |
| H0YGH4 | A2M | Alpha-2-macroglobulin | 5,27 | -0,195 | 6,03 | 163291 | 129 | 169 |
| P01024 | C3 | Complement C3 | 4,82 | -0,320 | 6,02 | 187148,06 | 134 | 163 |
| P02763 | ORM1 | Alpha-1-acid glycoprotein 1 | 4,21 | -0,536 | 4,93 | 23511,56 | 24 | 27 |
| P06727 | APOA4 | Apolipoprotein A-IV | 4,16 | -0,796 | 5,28 | 45399,06 | 20 | 28 |
| P01859 | IGHG2 | Ig gamma-2 chain C region | 3,91 | -0,419 | 7,66 | 35900,64 | 28 | 36 |
| P01860 | IGHG3 | Ig gamma-3 chain C region | 3,81 | -0,511 | 8,23 | 41286,96 | 27 | 35 |
| P02790 | HPX | Hemopexin | 3,72 | -0,434 | 6,55 | 51676,37 | 55 | 74 |
| B0V2C8 | C4A | Complement C4-A | 3,53 | -0,252 | 6,66 | 192785,48 | 139 | 177 |
| P02656 | APOC3 | Apolipoprotein C-III | 3,53 | -0,086 | 5,23 | 10852,31 | 9 | 10 |
| P02679-2 | FGG | Fibrinogen gamma chain | 3,51 | -0,588 | 5,37 | 51511,66 | 55 | 67 |

| **F1 HC** | Average SD |  | 5,42  0,91 | -0,477 0,225 | 6,25  0,98 | 80008,28  112130,65 | 62,05  90,00 | 78,85  114,60 |
| --- | --- | --- | --- | --- | --- | --- | --- | --- |
|  |  |  |  |  |  |  |  |  |
| **F1 SC** | Average SD |  | 4,15  1,24 | -0,439 0,214 | 6,50  1,01 | 63855,90  54531,47 | 48,55  40,87 | 62,10  51,62 |
|  |  |  |  |  |  |  |  |  |

| **F2 HC** | Average SD |  | 5,20  0,84 | -0,439 0,208 | 6,30  0,97 | 98278,34  121408,55 | 75,10  95,45 | 95,50  121,35 |
| --- | --- | --- | --- | --- | --- | --- | --- | --- |
|  |  |  |  |  |  |  |  |  |
| **F2 SC** | Average SD |  | 4,41  1,23 | -0,436 0,216 | 6,60  1,08 | 63298,82  54742,99 | 48,75  40,81 | 62,15  51,61 |
|  |  |  |  |  |  |  |  |  |

| **All HC** | **Average SD** | **3,84**  **1,75** | **-0,415 0,229** | **6,35**  **1,02** | **87064,66**  **111169,11** | **67,51**  **87,57** | **85,71**  **111,40** |
| --- | --- | --- | --- | --- | --- | --- | --- |
|  |  |  |  |  |  |  |  |
|  | |  |  |  |  |  |  |
| **All SC** | **Average SD** | **4,50**  **1,06** | **-0,447 0,212** | **6,43**  **1,07** | **60155,44**  **49847,93** | **46,49**  **38,31** | **59,63**  **48,35** |
|  |  |  |  |  |  |  |  |
|  | |  |  |  |  |  |  |
| **Source** | **Average SD** | **4,26**  **1,15** | **-0,434 0,203** | **6,35**  **1,07** | **62320,15**  **48874,87** | **48,15**  **39,34** | **61,30**  **48,74** |
|  |  |  |  |  |  |  |  |

| P01834 | IGKC | Ig kappa chain C region | 3,51 | -0,537 | 6,11 | 11765,04 | 9 | 11 |
| --- | --- | --- | --- | --- | --- | --- | --- | --- |
| F5H0Q5 | AHSG | Alpha-2-HS-glycoprotein | 3,26 | -0,190 | 5,43 | 39340,74 | 20 | 33 |
|  | **Average** |  | **4,62** | **-0,437** | **6,31** | **63945,71** | **48,65** | **62,50** |
|  | **SD** |  | **0,98** | **0,221** | **1,11** | **54188,71** | **40,55** | **51,02** |
| **F7** | **HC** |  |  |  |  |  |  |  |
| P02671 | FGA | Fibrinogen alpha chain | 5,26 | -0,822 | 5,7 | 94973,04 | 72 | 91 |
| P01024 | C3 | Complement C3 | 4,17 | -0,320 | 6,02 | 187148,06 | 134 | 163 |
| P01857 | IGHG1 | Ig gamma-1 chain C region | 4,17 | -0,428 | 8,46 | 36105,91 | 27 | 36 |
| P02675 | FGB | Fibrinogen beta chain | 4,08 | -0,758 | 8,54 | 55928,15 | 47 | 56 |
| P01766 | IGHV3-13 | Ig heavy chain V-III region BRO | 3,15 | 0,066 | 6,54 | 12506,22 | 13 | 14 |
| P10909-4 | CLU | Clusterin | 3,07 | -0,665 | 5,88 | 52494,58 | 39 | 52 |
| P01834 | IGKC | Ig kappa chain C region | 3,04 | -0,537 | 6,11 | 11765,04 | 9 | 11 |
| P00738 | HP | Haptoglobin | 2,89 | -0,421 | 6,13 | 45205,31 | 37 | 50 |
| P02787 | TF | Serotransferrin | 2,61 | -0,336 | 6,81 | 77063,89 | 62 | 81 |
| P01619 | IGKV3-20 | Ig kappa chain V-III region WOL | 2,55 | -0,130 | 4,85 | 12557,12 | 12 | 12 |
| H0YGH4 | A2M | Alpha-2-macroglobulin | 2,05 | -0,195 | 6,03 | 163291 | 129 | 169 |
| B0V2C8 | C4A | Complement C4-A | 1,87 | -0,252 | 6,66 | 192785,48 | 139 | 177 |
| P0CG05 | IGLC2 | Ig lambda-2 chain C regions | 1,76 | -0,444 | 6,91 | 11293,57 | 8 | 10 |
| F5H0Q5 | AHSG | Alpha-2-HS-glycoprotein | 1,37 | -0,190 | 5,43 | 39340,74 | 20 | 33 |
| P01764 | IGHV3-23 | Ig heavy chain V-III region TIL | 1,04 | 0,073 | 8,49 | 12582,31 | 14 | 14 |
| P01859 | IGHG2 | Ig gamma-2 chain C region | 1,01 | -0,419 | 7,66 | 35900,64 | 28 | 36 |
| P04114 | APOB | Apolipoprotein B-100 | 1,00 | -0,296 | 6,58 | 515604,73 | 414 | 528 |
| P02751-14 | FN1 | Fibronectin | 0,91 | -0,514 | 5,31 | 272320,36 | 198 | 249 |
| P04004 | VTN | Vitronectin | 0,50 | -0,723 | 5,55 | 54305,59 | 56 | 65 |
| P06312 | IGKV4-1 | Ig kappa chain V-IV region Len | 0,39 | -0,143 | 5,09 | 13380,05 | 15 | 15 |
|  | **Average** |  | **2,35** | **-0,373** | **6,44** | **94827,59** | **73,65** | **93,10** |
|  | **SD** |  | **1,39** | **0,252** | **1,11** | **123524,62** | **96,27** | **122,68** |
| **F7** | **SC** |  |  |  |  |  |  |  |
| P02647 | APOA1 | Apolipoprotein A-I | 6,02 | -0,717 | 5,56 | 30777,83 | 20 | 26 |
| P02787 | TF | Serotransferrin | 5,65 | -0,336 | 6,81 | 77063,89 | 62 | 81 |
| P01876 | IGHA1 | Ig alpha-1 chain C region | 5,64 | -0,206 | 6,08 | 37654,65 | 23 | 31 |
| P02671 | FGA | Fibrinogen alpha chain | 5,40 | -0,822 | 5,7 | 94973,04 | 72 | 91 |
| P01009 | SERPINA1 | Alpha-1-antitrypsin | 5,24 | -0,183 | 5,37 | 46736,55 | 36 | 49 |
| P01024 | C3 | Complement C3 | 5,12 | -0,320 | 6,02 | 187148,06 | 134 | 163 |
| P02675 | FGB | Fibrinogen beta chain | 5,04 | -0,758 | 8,54 | 55928,15 | 47 | 56 |
| P01857 | IGHG1 | Ig gamma-1 chain C region | 4,58 | -0,428 | 8,46 | 36105,91 | 27 | 36 |
| P0CG05 | IGLC2 | Ig lambda-2 chain C regions | 4,54 | -0,444 | 6,91 | 11293,57 | 8 | 10 |
| H0YGH4 | A2M | Alpha-2-macroglobulin | 4,49 | -0,195 | 6,03 | 163291 | 129 | 169 |
| P02763 | ORM1 | Alpha-1-acid glycoprotein 1 | 4,25 | -0,536 | 4,93 | 23511,56 | 24 | 27 |
| P01834 | IGKC | Ig kappa chain C region | 4,19 | -0,537 | 6,11 | 11765,04 | 9 | 11 |
| P06727 | APOA4 | Apolipoprotein A-IV | 4,06 | -0,796 | 5,28 | 45399,06 | 20 | 28 |
| F5H0Q5 | AHSG | Alpha-2-HS-glycoprotein | 3,96 | -0,190 | 5,43 | 39340,74 | 20 | 33 |
| P02679-2 | FGG | Fibrinogen gamma chain | 3,85 | -0,588 | 5,37 | 51511,66 | 55 | 67 |
| P02656 | APOC3 | Apolipoprotein C-III | 3,73 | -0,086 | 5,23 | 10852,31 | 9 | 10 |
| P01619 | IGKV3-20 | Ig kappa chain V-III region WOL | 3,66 | -0,130 | 4,85 | 12557,12 | 12 | 12 |
| P01766 | IGHV3-13 | Ig heavy chain V-III region BRO | 3,48 | 0,066 | 6,54 | 12506,22 | 13 | 14 |
| P02790 | HPX | Hemopexin | 3,36 | -0,434 | 6,55 | 51676,37 | 55 | 74 |
| P01860 | IGHG3 | Ig gamma-3 chain C region | 3,14 | -0,511 | 8,23 | 41286,96 | 27 | 35 |
|  | **Average** |  | **4,47** | **-0,408** | **6,20** | **52068,98** | **40,10** | **51,15** |
|  | **SD** |  | **0,84** | **0,254** | **1,12** | **47734,15** | **36,51** | **46,21** |
|  | |  | **Fold** | **GRAVY** | **pI** | **MW** | **F+Y+W** | **F+Y+W+H** |
| **All HC+SC Average** | |  | **4,18** | **-0,431** | **6,39** | **72793,49** | **56,36** | **71,85** |
| **SD** | |  | **1,46** | **0,219** | **1,04** | **84875,06** | **66,62** | **84,55** |

| **F3 HC** | Average SD | 5,28  0,95 | -0,450 0,217 | 6,28  0,98 | 80345,20  112012,63 | 62,20  89,90 | 79,80  114,26 |
| --- | --- | --- | --- | --- | --- | --- | --- |
|  |  |  |  |  |  |  |  |
| **F3 SC** | Average SD | 4,05  1,02 | -0,477 0,205 | 6,52  1,09 | 60218,69  47872,95 | 46,41  36,78 | 59,59  46,15 |
|  |  |  |  |  |  |  |  |
| **F4 HC** | Average SD | 2,31  1,30 | -0,391 0,244 | 6,56  1,16 | 91856,84  120654,22 | 72,45  94,55 | 91,50  120,40 |
|  |  |  |  |  |  |  |  |
| **F4 SC** | Average SD | 4,79  0,96 | -0,465 0,174 | 6,49  1,06 | 57328,87  49345,16 | 47,20  41,19 | 60,40  52,61 |
|  |  |  |  |  |  |  |  |
| **F5 HC** | Average SD | 2,75  1,31 | -0,361 0,215 | 6,33  1,01 | 98639,95  122409,93 | 76,45  95,68 | 96,95  121,77 |
|  |  |  |  |  |  |  |  |
| **F5 SC** | Average SD | 4,95  0,90 | -0,473 0,219 | 6,40  1,13 | 60380,59  46026,88 | 45,75  36,06 | 59,50  44,77 |
|  |  |  |  |  |  |  |  |
| **F6 HC** | Average SD | 3,48  1,20 | -0,416 0,251 | 6,29  1,00 | 60104,36  51301,20 | 46,50  38,74 | 58,94  48,74 |
|  |  |  |  |  |  |  |  |
| **F6 SC** | Average SD | 4,62  0,98 | -0,437 0,221 | 6,31  1,11 | 63945,71  54188,71 | 48,65  40,55 | 62,50  51,02 |
|  |  |  |  |  |  |  |  |
| **F7 HC** | Average SD | 2,35  1,39 | -0,373 0,252 | 6,44  1,11 | 94827,59  123524,62 | 73,65  96,27 | 93,10  122,68 |
|  |  |  |  |  |  |  |  |
| **F7 SC** | Average SD | 4,47  0,84 | -0,408 0,254 | 6,20  1,12 | 52068,98  47734,15 | 40,10  36,51 | 51,15  46,21 |
|  |  |  |  |  |  |  |  |

**Supplementary File 4**

**Top 20 VITREOUS**

**VITREOUS Top20 enriched**

| **Source** |  | **Protein names** | **Fold** | **GRAVY** | **pI** | **MW** | **F+Y+W** |  | **F+Y+W+H** |
| --- | --- | --- | --- | --- | --- | --- | --- | --- | --- |
| P02787 | TF | Serotransferrin | 6,54 | -0,336 | 6,81 | 77063,89 |  | 62 | 81 |
| P01876 | IGHA1 | Ig alpha-1 chain C region | 5,96 | -0,206 | 6,08 | 37654,65 |  | 23 | 31 |
| P00738 | HP | Haptoglobin | 5,96 | -0,421 | 6,13 | 45205,31 |  | 37 | 50 |
| P01834 | IGKC | Ig kappa chain C region | 5,89 | -0,537 | 6,11 | 11765,04 |  | 9 | 11 |
| P0CG05 | IGLC2 | Ig lambda-2 chain C regions | 5,41 | -0,444 | 6,91 | 11293,57 |  | 8 | 10 |
| P02675 | FGB | Fibrinogen beta chain | 5,07 | -0,758 | 8,54 | 55928,15 |  | 47 | 56 |
| P02656 | APOC3 | Apolipoprotein C-III | 4,45 | -0,086 | 5,23 | 10852,31 |  | 9 | 10 |
| P01857 | IGHG1 | Ig gamma-1 chain C region | 4,24 | -0,428 | 8,46 | 36105,91 |  | 27 | 36 |
| F5H0Q5 | AHSG | Alpha-2-HS-glycoprotein | 4,12 | -0,190 | 5,43 | 39340,74 |  | 20 | 33 |
| P02774 | GC | Vitamin D-binding protein | 3,97 | -0,336 | 5,32 | 52917,54 |  | 36 | 44 |
| P02647 | APOA1 | Apolipoprotein A-I | 3,94 | -0,717 | 5,56 | 30777,83 |  | 20 | 26 |
| P01024 | C3 | Complement C3 | 3,85 | -0,320 | 6,02 | 187148,06 |  | 134 | 163 |
| P02671 | FGA | Fibrinogen alpha chain | 3,78 | -0,822 | 5,7 | 94973,04 |  | 72 | 91 |
| P04217 | A1BG | Alpha-1B-glycoprotein | 3,40 | -0,217 | 5,56 | 54253,52 |  | 38 | 54 |
| P00734 | F2 | Prothrombin | 3,38 | -0,539 | 5,63 | 70036,87 |  | 56 | 69 |
| H0YGH4 | A2M | Alpha-2-macroglobulin | 3,18 | -0,195 | 6,03 | 163291 |  | 129 | 169 |
| P01859 | IGHG2 | Ig gamma-2 chain C region | 3,17 | -0,419 | 7,66 | 35900,64 |  | 28 | 36 |
| P02679-2 | FGG | Fibrinogen gamma chain | 3,10 | -0,588 | 5,37 | 51511,66 |  | 55 | 67 |
| P01860 | IGHG3 | Ig gamma-3 chain C region | 2,90 | -0,511 | 8,23 | 41286,96 |  | 27 | 35 |
| P08603 | CFH | Complement factor H | 2,87 | -0,603 | 6,21 | 139096,31 |  | 126 | 154 |
|  | **Average** |  | **4,26** | **-0,434** | **6,35** | **62320,15** |  | **48,15** | **61,30** |
|  | **SD** |  | **1,15** | **0,203** | **1,07** | **48874,87** |  | **39,34** | **48,74** |
| **F1** | **HC** |  |  |  |  |  |  |  |  |
| P10909-4 | CLU | Clusterin | 7,32 | -0,665 | 5,88 | 52494,58 |  | 39 | 52 |
| P02787 | TF | Serotransferrin | 6,79 | -0,336 | 6,81 | 77063,89 |  | 62 | 81 |
| P02679-2 | FGG | Fibrinogen gamma chain | 6,45 | -0,588 | 5,37 | 51511,66 |  | 55 | 67 |
| P01857 | IGHG1 | Ig gamma-1 chain C region | 6,31 | -0,428 | 8,46 | 36105,91 |  | 27 | 36 |
| P02647 | APOA1 | Apolipoprotein A-I | 5,98 | -0,717 | 5,56 | 30777,83 |  | 20 | 26 |
| P02671 | FGA | Fibrinogen alpha chain | 5,74 | -0,822 | 5,7 | 94973,04 |  | 72 | 91 |
| P02649 | APOE | Apolipoprotein E | 5,73 | -0,596 | 5,65 | 36154,08 |  | 16 | 18 |
| P01834 | IGKC | Ig kappa chain C region | 5,68 | -0,537 | 6,11 | 11765,04 |  | 9 | 11 |
| P01024 | C3 | Complement C3 | 5,60 | -0,320 | 6,02 | 187148,06 |  | 134 | 163 |
| P00738 | HP | Haptoglobin | 5,57 | -0,421 | 6,13 | 45205,31 |  | 37 | 50 |
| P06727 | APOA4 | Apolipoprotein A-IV | 5,41 | -0,796 | 5,28 | 45399,06 |  | 20 | 28 |
| P04114 | APOB | Apolipoprotein B-100 | 5,37 | -0,296 | 6,58 | 515604,73 |  | 414 | 528 |
| P02656 | APOC3 | Apolipoprotein C-III | 5,30 | -0,086 | 5,23 | 10852,31 |  | 9 | 10 |
| P0CG05 | IGLC2 | Ig lambda-2 chain C regions | 5,01 | -0,444 | 6,91 | 11293,57 |  | 8 | 10 |
| H0YGH4 | A2M | Alpha-2-macroglobulin | 4,95 | -0,195 | 6,03 | 163291 |  | 129 | 169 |
| P01009 | SERPINA1 | Alpha-1-antitrypsin | 4,49 | -0,183 | 5,37 | 46736,55 |  | 36 | 49 |
| P04004 | VTN | Vitronectin | 4,29 | -0,723 | 5,55 | 54305,59 |  | 56 | 65 |
| P02675 | FGB | Fibrinogen beta chain | 4,28 | -0,758 | 8,54 | 55928,15 |  | 47 | 56 |
| P01876 | IGHA1 | Ig alpha-1 chain C region | 4,03 | -0,206 | 6,08 | 37654,65 |  | 23 | 31 |
| P01859 | IGHG2 | Ig gamma-2 chain C region | 4,00 | -0,419 | 7,66 | 35900,64 |  | 28 | 36 |
|  | **Average** |  | **5,42** | **-0,477** | **6,25** | **80008,28** |  | **62,05** | **78,85** |
|  | **SD** |  | **0,91** | **0,225** | **0,98** | **112130,65** |  | **90,00** | **114,60** |
| **F1** | **SC** |  |  |  |  |  |  |  |  |
| P02787 | TF | Serotransferrin | 6,29 | -0,336 | 6,81 | 77063,89 |  | 62 | 81 |
| P01857 | IGHG1 | Ig gamma-1 chain C region | 5,91 | -0,428 | 8,46 | 36105,91 |  | 27 | 36 |
| P02647 | APOA1 | Apolipoprotein A-I | 5,57 | -0,717 | 5,56 | 30777,83 |  | 20 | 26 |
| P01834 | IGKC | Ig kappa chain C region | 5,41 | -0,537 | 6,11 | 11765,04 |  | 9 | 11 |
| H0YGH4 | A2M | Alpha-2-macroglobulin | 5,04 | -0,195 | 6,03 | 163291 |  | 129 | 169 |
| P00738 | HP | Haptoglobin | 5,02 | -0,421 | 6,13 | 45205,31 |  | 37 | 50 |
| P01876 | IGHA1 | Ig alpha-1 chain C region | 4,59 | -0,206 | 6,08 | 37654,65 |  | 23 | 31 |
| P01024 | C3 | Complement C3 | 4,59 | -0,320 | 6,02 | 187148,06 |  | 134 | 163 |
| P02679-2 | FGG | Fibrinogen gamma chain | 4,50 | -0,588 | 5,37 | 51511,66 |  | 55 | 67 |
| P0CG05 | IGLC2 | Ig lambda-2 chain C regions | 4,50 | -0,444 | 6,91 | 11293,57 |  | 8 | 10 |
| P02675 | FGB | Fibrinogen beta chain | 4,45 | -0,758 | 8,54 | 55928,15 |  | 47 | 56 |
| P01009 | SERPINA1 | Alpha-1-antitrypsin | 4,12 | -0,183 | 5,37 | 46736,55 |  | 36 | 49 |
| P02671 | FGA | Fibrinogen alpha chain | 3,87 | -0,822 | 5,7 | 94973,04 |  | 72 | 91 |
| P02790 | HPX | Hemopexin | 3,39 | -0,434 | 6,55 | 51676,37 |  | 55 | 74 |
| P01859 | IGHG2 | Ig gamma-2 chain C region | 3,32 | -0,419 | 7,66 | 35900,64 |  | 28 | 36 |
| B0V2C8 | C4A | Complement C4-A | 2,80 | -0,252 | 6,66 | 192785,48 |  | 139 | 177 |
| P01860 | IGHG3 | Ig gamma-3 chain C region | 2,66 | -0,511 | 8,23 | 41286,96 |  | 27 | 35 |
| P01871 | IGHM | Ig mu chain C region | 2,36 | -0,319 | 6,35 | 49439,74 |  | 35 | 44 |
| P06727 | APOA4 | Apolipoprotein A-IV | 2,34 | -0,796 | 5,28 | 45399,06 |  | 20 | 28 |
| P02652 | APOA2 | Apolipoprotein A-II | 2,25 | -0,084 | 6,27 | 11175,02 |  | 8 | 8 |
|  | **Average** |  | **4,15** | **-0,439** | **6,50** | **63855,90** |  | **48,55** | **62,10** |
|  | **SD** |  | **1,24** | **0,214** | **1,01** | **54531,47** |  | **40,87** | **51,62** |
| **F2** | **HC** |  |  |  |  |  |  |  |  |
| P02787 | TF | Serotransferrin | 6,78 | -0,336 | 6,81 | 77063,89 |  | 62 | 81 |
| P10909-4 | CLU | Clusterin | 6,59 | -0,665 | 5,88 | 52494,58 |  | 39 | 52 |

| P01857 | IGHG1 | Ig gamma-1 chain C region | 6,46 | -0,428 | 8,46 | 36105,91 | 27 | 36 |
| --- | --- | --- | --- | --- | --- | --- | --- | --- |
| P01834 | IGKC | Ig kappa chain C region | 5,86 | -0,537 | 6,11 | 11765,04 | 9 | 11 |
| H0YGH4 | A2M | Alpha-2-macroglobulin | 5,69 | -0,195 | 6,03 | 163291 | 129 | 169 |
| P02671 | FGA | Fibrinogen alpha chain | 5,64 | -0,822 | 5,7 | 94973,04 | 72 | 91 |
| P01024 | C3 | Complement C3 | 5,55 | -0,320 | 6,02 | 187148,06 | 134 | 163 |
| P0CG05 | IGLC2 | Ig lambda-2 chain C regions | 5,53 | -0,444 | 6,91 | 11293,57 | 8 | 10 |
| P02675 | FGB | Fibrinogen beta chain | 5,31 | -0,758 | 8,54 | 55928,15 | 47 | 56 |
| P01876 | IGHA1 | Ig alpha-1 chain C region | 5,24 | -0,206 | 6,08 | 37654,65 | 23 | 31 |
| P02649 | APOE | Apolipoprotein E | 5,23 | -0,596 | 5,65 | 36154,08 | 16 | 18 |
| P02679-2 | FGG | Fibrinogen gamma chain | 4,91 | -0,588 | 5,37 | 51511,66 | 55 | 67 |
| P02647 | APOA1 | Apolipoprotein A-I | 4,88 | -0,717 | 5,56 | 30777,83 | 20 | 26 |
| P01009 | SERPINA1 | Alpha-1-antitrypsin | 4,83 | -0,183 | 5,37 | 46736,55 | 36 | 49 |
| P02656 | APOC3 | Apolipoprotein C-III | 4,69 | -0,086 | 5,23 | 10852,31 | 9 | 10 |
| P04114 | APOB | Apolipoprotein B-100 | 4,45 | -0,296 | 6,58 | 515604,73 | 414 | 528 |
| B0V2C8 | C4A | Complement C4-A | 4,43 | -0,252 | 6,66 | 192785,48 | 139 | 177 |
| P02751-14 | FN1 | Fibronectin | 4,11 | -0,514 | 5,31 | 272320,36 | 198 | 249 |
| P00738 | HP | Haptoglobin | 3,93 | -0,421 | 6,13 | 45205,31 | 37 | 50 |
| P01859 | IGHG2 | Ig gamma-2 chain C region | 3,85 | -0,419 | 7,66 | 35900,64 | 28 | 36 |
|  | **Average** |  | **5,20** | **-0,439** | **6,30** | **98278,34** | **75,10** | **95,50** |
|  | **SD** |  | **0,84** | **0,208** | **0,97** | **121408,55** | **95,45** | **121,35** |
| **F2** | **SC** |  |  |  |  |  |  |  |
| P02787 | TF | Serotransferrin | 6,55 | -0,336 | 6,81 | 77063,89 | 62 | 81 |
| P01857 | IGHG1 | Ig gamma-1 chain C region | 6,39 | -0,428 | 8,46 | 36105,91 | 27 | 36 |
| P02647 | APOA1 | Apolipoprotein A-I | 5,95 | -0,717 | 5,56 | 30777,83 | 20 | 26 |
| P01834 | IGKC | Ig kappa chain C region | 5,83 | -0,537 | 6,11 | 11765,04 | 9 | 11 |
| P00738 | HP | Haptoglobin | 5,30 | -0,421 | 6,13 | 45205,31 | 37 | 50 |
| H0YGH4 | A2M | Alpha-2-macroglobulin | 5,09 | -0,195 | 6,03 | 163291 | 129 | 169 |
| P01024 | C3 | Complement C3 | 5,01 | -0,320 | 6,02 | 187148,06 | 134 | 163 |
| P01876 | IGHA1 | Ig alpha-1 chain C region | 4,82 | -0,206 | 6,08 | 37654,65 | 23 | 31 |
| P02679-2 | FGG | Fibrinogen gamma chain | 4,81 | -0,588 | 5,37 | 51511,66 | 55 | 67 |
| P0CG05 | IGLC2 | Ig lambda-2 chain C regions | 4,61 | -0,444 | 6,91 | 11293,57 | 8 | 10 |
| P02675 | FGB | Fibrinogen beta chain | 4,60 | -0,758 | 8,54 | 55928,15 | 47 | 56 |
| P02671 | FGA | Fibrinogen alpha chain | 4,28 | -0,822 | 5,7 | 94973,04 | 72 | 91 |
| P02790 | HPX | Hemopexin | 3,66 | -0,434 | 6,55 | 51676,37 | 55 | 74 |
| P01859 | IGHG2 | Ig gamma-2 chain C region | 3,56 | -0,419 | 7,66 | 35900,64 | 28 | 36 |
| P01009 | SERPINA1 | Alpha-1-antitrypsin | 3,26 | -0,183 | 5,37 | 46736,55 | 36 | 49 |
| B0V2C8 | C4A | Complement C4-A | 3,07 | -0,252 | 6,66 | 192785,48 | 139 | 177 |
| P02652 | APOA2 | Apolipoprotein A-II | 3,07 | -0,084 | 6,27 | 11175,02 | 8 | 8 |
| P01860 | IGHG3 | Ig gamma-3 chain C region | 2,93 | -0,511 | 8,23 | 41286,96 | 27 | 35 |
| P06727 | APOA4 | Apolipoprotein A-IV | 2,89 | -0,796 | 5,28 | 45399,06 | 20 | 28 |
| P02749 | APOH | Beta-2-glycoprotein 1 | 2,62 | -0,274 | 8,34 | 38298,16 | 39 | 45 |
|  | **Average** |  | **4,41** | **-0,436** | **6,60** | **63298,82** | **48,75** | **62,15** |
|  | **SD** |  | **1,23** | **0,216** | **1,08** | **54742,99** | **40,81** | **51,61** |
| **F3** | **HC** |  |  |  |  |  |  |  |
| P02787 | TF | Serotransferrin | 7,13 | -0,336 | 6,81 | 77063,89 | 62 | 81 |
| P01857 | IGHG1 | Ig gamma-1 chain C region | 6,43 | -0,428 | 8,46 | 36105,91 | 27 | 36 |
| P01834 | IGKC | Ig kappa chain C region | 6,36 | -0,537 | 6,11 | 11765,04 | 9 | 11 |
| H0YGH4 | A2M | Alpha-2-macroglobulin | 5,96 | -0,195 | 6,03 | 163291 | 129 | 169 |
| P00738 | HP | Haptoglobin | 5,92 | -0,421 | 6,13 | 45205,31 | 37 | 50 |
| P0CG05 | IGLC2 | Ig lambda-2 chain C regions | 5,92 | -0,444 | 6,91 | 11293,57 | 8 | 10 |
| P01024 | C3 | Complement C3 | 5,76 | -0,320 | 6,02 | 187148,06 | 134 | 163 |
| P02679-2 | FGG | Fibrinogen gamma chain | 5,72 | -0,588 | 5,37 | 51511,66 | 55 | 67 |
| P10909-4 | CLU | Clusterin | 5,64 | -0,665 | 5,88 | 52494,58 | 39 | 52 |
| P01876 | IGHA1 | Ig alpha-1 chain C region | 5,52 | -0,206 | 6,08 | 37654,65 | 23 | 31 |
| P02671 | FGA | Fibrinogen alpha chain | 5,49 | -0,822 | 5,7 | 94973,04 | 72 | 91 |
| P02675 | FGB | Fibrinogen beta chain | 5,40 | -0,758 | 8,54 | 55928,15 | 47 | 56 |
| P02647 | APOA1 | Apolipoprotein A-I | 4,99 | -0,717 | 5,56 | 30777,83 | 20 | 26 |
| P06727 | APOA4 | Apolipoprotein A-IV | 4,97 | -0,796 | 5,28 | 45399,06 | 20 | 28 |
| F5H0Q5 | AHSG | Alpha-2-HS-glycoprotein | 4,45 | -0,190 | 5,43 | 39340,74 | 20 | 33 |
| P01859 | IGHG2 | Ig gamma-2 chain C region | 4,18 | -0,419 | 7,66 | 35900,64 | 28 | 36 |
| P04114 | APOB | Apolipoprotein B-100 | 4,15 | -0,296 | 6,58 | 515604,73 | 414 | 528 |
| P02774 | GC | Vitamin D-binding protein | 4,06 | -0,336 | 5,32 | 52917,54 | 36 | 44 |
| P02790 | HPX | Hemopexin | 3,94 | -0,434 | 6,55 | 51676,37 | 55 | 74 |
| P02656 | APOC3 | Apolipoprotein C-III | 3,60 | -0,086 | 5,23 | 10852,31 | 9 | 10 |
|  | **Average** |  | **5,28** | **-0,450** | **6,28** | **80345,20** | **62,20** | **79,80** |
|  | **SD** |  | **0,95** | **0,217** | **0,98** | **112012,63** | **89,90** | **114,26** |
| **F3** | **SC** |  |  |  |  |  |  |  |
| P02787 | TF | Serotransferrin | 5,87 | -0,336 | 6,81 | 77063,89 | 62 | 81 |
| P01857 | IGHG1 | Ig gamma-1 chain C region | 5,58 | -0,428 | 8,46 | 36105,91 | 27 | 36 |
| P02647 | APOA1 | Apolipoprotein A-I | 5,20 | -0,717 | 5,56 | 30777,83 | 20 | 26 |
| P01834 | IGKC | Ig kappa chain C region | 4,96 | -0,537 | 6,11 | 11765,04 | 9 | 11 |
| P00738 | HP | Haptoglobin | 4,70 | -0,421 | 6,13 | 45205,31 | 37 | 50 |
| H0YGH4 | A2M | Alpha-2-macroglobulin | 4,60 | -0,195 | 6,03 | 163291 | 129 | 169 |

| P01876 | IGHA1 | Ig alpha-1 chain C region | 4,20 | -0,206 | 6,08 | 37654,65 | 23 | 31 |
| --- | --- | --- | --- | --- | --- | --- | --- | --- |
| P01024 | C3 | Complement C3 | 4,07 | -0,320 | 6,02 | 187148,06 | 134 | 163 |
| P0CG05 | IGLC2 | Ig lambda-2 chain C regions | 4,02 | -0,444 | 6,91 | 11293,57 | 8 | 10 |
| P01859 | IGHG2 | Ig gamma-2 chain C region | 3,90 | -0,419 | 7,66 | 35900,64 | 28 | 36 |
| P02671 | FGA | Fibrinogen alpha chain | 3,66 | -0,822 | 5,7 | 94973,04 | 72 | 91 |
| P02679-2 | FGG | Fibrinogen gamma chain | 3,58 | -0,588 | 5,37 | 51511,66 | 55 | 67 |
| P06727 | APOA4 | Apolipoprotein A-IV | 3,44 | -0,796 | 5,28 | 45399,06 | 20 | 28 |
| P01009 | SERPINA1 | Alpha-1-antitrypsin | 3,30 | -0,183 | 5,37 | 46736,55 | 36 | 49 |
| P02675 | FGB | Fibrinogen beta chain | 2,87 | -0,758 | 8,54 | 55928,15 | 47 | 56 |
| P02790 | HPX | Hemopexin | 2,86 | -0,434 | 6,55 | 51676,37 | 55 | 74 |
| P01860 | IGHG3 | Ig gamma-3 chain C region | 2,06 | -0,511 | 8,23 | 41286,96 | 27 | 35 |
|  | **Average** |  | **4,05** | **-0,477** | **6,52** | **60218,69** | **46,41** | **59,59** |
|  | **SD** |  | **1,02** | **0,205** | **1,09** | **47872,95** | **36,78** | **46,15** |
| **F4** | **HC** |  |  |  |  |  |  |  |
| P01024 | C3 | Complement C3 | 4,61 | -0,320 | 6,02 | 187148,06 | 134 | 163 |
| P01857 | IGHG1 | Ig gamma-1 chain C region | 4,35 | -0,428 | 8,46 | 36105,91 | 27 | 36 |
| P02675 | FGB | Fibrinogen beta chain | 4,12 | -0,758 | 8,54 | 55928,15 | 47 | 56 |
| P00738 | HP | Haptoglobin | 3,44 | -0,421 | 6,13 | 45205,31 | 37 | 50 |
| P02787 | TF | Serotransferrin | 3,36 | -0,336 | 6,81 | 77063,89 | 62 | 81 |
| P01834 | IGKC | Ig kappa chain C region | 3,15 | -0,537 | 6,11 | 11765,04 | 9 | 11 |
| P01619 | IGKV3-20 | Ig kappa chain V-III region WOL | 2,97 | -0,130 | 4,85 | 12557,12 | 12 | 12 |
| H0YGH4 | A2M | Alpha-2-macroglobulin | 2,97 | -0,195 | 6,03 | 163291 | 129 | 169 |
| P01766 | IGHV3-13 | Ig heavy chain V-III region BRO | 2,87 | 0,066 | 6,54 | 12506,22 | 13 | 14 |
| P02671 | FGA | Fibrinogen alpha chain | 2,77 | -0,822 | 5,7 | 94973,04 | 72 | 91 |
| P02751-14 | FN1 | Fibronectin | 2,03 | -0,514 | 5,31 | 272320,36 | 198 | 249 |
| P02679-2 | FGG | Fibrinogen gamma chain | 1,66 | -0,588 | 5,37 | 51511,66 | 55 | 67 |
| P04004 | VTN | Vitronectin | 1,34 | -0,723 | 5,55 | 54305,59 | 56 | 65 |
| P01764 | IGHV3-23 | Ig heavy chain V-III region TIL | 1,13 | 0,073 | 8,49 | 12582,31 | 14 | 14 |
| F5H0Q5 | AHSG | Alpha-2-HS-glycoprotein | 1,09 | -0,190 | 5,43 | 39340,74 | 20 | 33 |
| Q5T985 | ITIH2 | Inter-alpha-trypsin inhibitor heavy chain H2 | 1,05 | -0,293 | 6,4 | 106463,47 | 87 | 110 |
| P04114 | APOB | Apolipoprotein B-100 | 1,02 | -0,296 | 6,58 | 515604,73 | 414 | 528 |
| P01860 | IGHG3 | Ig gamma-3 chain C region | 0,86 | -0,511 | 8,23 | 41286,96 | 27 | 35 |
| P01859 | IGHG2 | Ig gamma-2 chain C region | 0,76 | -0,419 | 7,66 | 35900,64 | 28 | 36 |
| P0CF74 | IGLC6 | Ig lambda-6 chain C region | 0,64 | -0,467 | 6,91 | 11276,54 | 8 | 10 |
|  | **Average** |  | **2,31** | **-0,391** | **6,56** | **91856,84** | **72,45** | **91,50** |
|  | **SD** |  | **1,30** | **0,244** | **1,16** | **120654,22** | **94,55** | **120,40** |
| **F4** | **SC** |  |  |  |  |  |  |  |
| H0YGH4 | A2M | Alpha-2-macroglobulin | 6,44 | -0,195 | 6,03 | 163291 | 129 | 169 |
| P02787 | TF | Serotransferrin | 6,35 | -0,336 | 6,81 | 77063,89 | 62 | 81 |
| P01857 | IGHG1 | Ig gamma-1 chain C region | 6,08 | -0,428 | 8,46 | 36105,91 | 27 | 36 |
| P01009 | SERPINA1 | Alpha-1-antitrypsin | 5,71 | -0,183 | 5,37 | 46736,55 | 36 | 49 |
| P01024 | C3 | Complement C3 | 5,46 | -0,320 | 6,02 | 187148,06 | 134 | 163 |
| P00738 | HP | Haptoglobin | 5,23 | -0,421 | 6,13 | 45205,31 | 37 | 50 |
| P02679-2 | FGG | Fibrinogen gamma chain | 5,21 | -0,588 | 5,37 | 51511,66 | 55 | 67 |
| P01876 | IGHA1 | Ig alpha-1 chain C region | 5,17 | -0,206 | 6,08 | 37654,65 | 23 | 31 |
| P02675 | FGB | Fibrinogen beta chain | 5,10 | -0,758 | 8,54 | 55928,15 | 47 | 56 |
| P01834 | IGKC | Ig kappa chain C region | 5,03 | -0,537 | 6,11 | 11765,04 | 9 | 11 |
| P01860 | IGHG3 | Ig gamma-3 chain C region | 4,98 | -0,511 | 8,23 | 41286,96 | 27 | 35 |
| P02671 | FGA | Fibrinogen alpha chain | 4,79 | -0,822 | 5,7 | 94973,04 | 72 | 91 |
| P02647 | APOA1 | Apolipoprotein A-I | 4,50 | -0,717 | 5,56 | 30777,83 | 20 | 26 |
| P02790 | HPX | Hemopexin | 4,03 | -0,434 | 6,55 | 51676,37 | 55 | 74 |
| P0CF74 | IGLC6 | Ig lambda-6 chain C region | 3,75 | -0,467 | 6,91 | 11276,54 | 8 | 10 |
| P0CG05 | IGLC2 | Ig lambda-2 chain C regions | 3,75 | -0,444 | 6,91 | 11293,57 | 8 | 10 |
| P0CG06 | IGLC3 | Ig lambda-3 chain C regions | 3,75 | -0,439 | 6,91 | 11265,56 | 8 | 10 |
| P01859 | IGHG2 | Ig gamma-2 chain C region | 3,73 | -0,419 | 7,66 | 35900,64 | 28 | 36 |
| P02763 | ORM1 | Alpha-1-acid glycoprotein 1 | 3,43 | -0,536 | 4,93 | 23511,56 | 24 | 27 |
| P00450 | CP | Ceruloplasmin | 3,39 | -0,536 | 5,44 | 122205,19 | 135 | 176 |
|  | **Average** |  | **4,79** | **-0,465** | **6,49** | **57328,87** | **47,20** | **60,40** |
|  | **SD** |  | **0,96** | **0,174** | **1,06** | **49345,16** | **41,19** | **52,61** |
| **F5** | **HC** |  |  |  |  |  |  |  |
| P01857 | IGHG1 | Ig gamma-1 chain C region | 4,69 | -0,428 | 8,46 | 36105,91 | 27 | 36 |
| P02671 | FGA | Fibrinogen alpha chain | 4,66 | -0,822 | 5,7 | 94973,04 | 72 | 91 |
| P01024 | C3 | Complement C3 | 4,61 | -0,320 | 6,02 | 187148,06 | 134 | 163 |
| P01619 | IGKV3-20 | Ig kappa chain V-III region WOL | 4,25 | -0,130 | 4,85 | 12557,12 | 12 | 12 |
| P02675 | FGB | Fibrinogen beta chain | 4,11 | -0,758 | 8,54 | 55928,15 | 47 | 56 |
| P00738 | HP | Haptoglobin | 3,96 | -0,421 | 6,13 | 45205,31 | 37 | 50 |
| P02787 | TF | Serotransferrin | 3,71 | -0,336 | 6,81 | 77063,89 | 62 | 81 |
| P01834 | IGKC | Ig kappa chain C region | 3,20 | -0,537 | 6,11 | 11765,04 | 9 | 11 |
| H0YGH4 | A2M | Alpha-2-macroglobulin | 3,14 | -0,195 | 6,03 | 163291 | 129 | 169 |
| P06312 | IGKV4-1 | Ig kappa chain V-IV region Len | 2,74 | -0,143 | 5,09 | 13380,05 | 15 | 15 |
| P01766 | IGHV3-13 | Ig heavy chain V-III region BRO | 2,17 | 0,066 | 6,54 | 12506,22 | 13 | 14 |
| P0CG05 | IGLC2 | Ig lambda-2 chain C regions | 2,05 | -0,444 | 6,91 | 11293,57 | 8 | 10 |
| B0V2C8 | C4A | Complement C4-A | 1,93 | -0,252 | 6,66 | 192785,48 | 139 | 177 |

| P02751-14 | FN1 | Fibronectin | 1,88 | -0,514 | 5,31 | 272320,36 | 198 | 249 |
| --- | --- | --- | --- | --- | --- | --- | --- | --- |
| F5H0Q5 | AHSG | Alpha-2-HS-glycoprotein | 1,71 | -0,190 | 5,43 | 39340,74 | 20 | 33 |
| P01859 | IGHG2 | Ig gamma-2 chain C region | 1,41 | -0,419 | 7,66 | 35900,64 | 28 | 36 |
| P04114 | APOB | Apolipoprotein B-100 | 1,32 | -0,296 | 6,58 | 515604,73 | 414 | 528 |
| P02679-2 | FGG | Fibrinogen gamma chain | 1,24 | -0,588 | 5,37 | 51511,66 | 55 | 67 |
| Q5T985 | ITIH2 | Inter-alpha-trypsin inhibitor heavy chain H2 | 1,12 | -0,293 | 6,4 | 106463,47 | 87 | 110 |
| P01876 | IGHA1 | Ig alpha-1 chain C region | 1,10 | -0,206 | 6,08 | 37654,65 | 23 | 31 |
|  | **Average** |  | **2,75** | **-0,361** | **6,33** | **98639,95** | **76,45** | **96,95** |
|  | **SD** |  | **1,31** | **0,215** | **1,01** | **122409,93** | **95,68** | **121,77** |
| **F5** | **SC** |  |  |  |  |  |  |  |
| P01857 | IGHG1 | Ig gamma-1 chain C region | 6,32 | -0,428 | 8,46 | 36105,91 | 27 | 36 |
| P02647 | APOA1 | Apolipoprotein A-I | 6,23 | -0,717 | 5,56 | 30777,83 | 20 | 26 |
| P02787 | TF | Serotransferrin | 6,04 | -0,336 | 6,81 | 77063,89 | 62 | 81 |
| P02671 | FGA | Fibrinogen alpha chain | 5,66 | -0,822 | 5,7 | 94973,04 | 72 | 91 |
| H0YGH4 | A2M | Alpha-2-macroglobulin | 5,55 | -0,195 | 6,03 | 163291 | 129 | 169 |
| P01024 | C3 | Complement C3 | 5,54 | -0,320 | 6,02 | 187148,06 | 134 | 163 |
| P00738 | HP | Haptoglobin | 5,53 | -0,421 | 6,13 | 45205,31 | 37 | 50 |
| P01009 | SERPINA1 | Alpha-1-antitrypsin | 5,49 | -0,183 | 5,37 | 46736,55 | 36 | 49 |
| P02675 | FGB | Fibrinogen beta chain | 5,31 | -0,758 | 8,54 | 55928,15 | 47 | 56 |
| P01876 | IGHA1 | Ig alpha-1 chain C region | 5,30 | -0,206 | 6,08 | 37654,65 | 23 | 31 |
| Q5T985 | ITIH2 | Inter-alpha-trypsin inhibitor heavy chain H2 | 5,30 | -0,293 | 6,4 | 106463,47 | 87 | 110 |
| P0CG05 | IGLC2 | Ig lambda-2 chain C regions | 4,91 | -0,444 | 6,91 | 11293,57 | 8 | 10 |
| P01834 | IGKC | Ig kappa chain C region | 4,53 | -0,537 | 6,11 | 11765,04 | 9 | 11 |
| Q9BUG6 | ZSCAN5A | Zinc finger and SCAN domain-containing protein 5A | 4,39 | -0,827 | 8,7 | 55865,34 | 22 | 42 |
| P02790 | HPX | Hemopexin | 4,05 | -0,434 | 6,55 | 51676,37 | 55 | 74 |
| P02679-2 | FGG | Fibrinogen gamma chain | 4,01 | -0,588 | 5,37 | 51511,66 | 55 | 67 |
| F5H0Q5 | AHSG | Alpha-2-HS-glycoprotein | 3,83 | -0,190 | 5,43 | 39340,74 | 20 | 33 |
| P02763 | ORM1 | Alpha-1-acid glycoprotein 1 | 3,71 | -0,536 | 4,93 | 23511,56 | 24 | 27 |
| P01859 | IGHG2 | Ig gamma-2 chain C region | 3,71 | -0,419 | 7,66 | 35900,64 | 28 | 36 |
| P06727 | APOA4 | Apolipoprotein A-IV | 3,57 | -0,796 | 5,28 | 45399,06 | 20 | 28 |
|  | **Average** |  | **4,95** | **-0,473** | **6,40** | **60380,59** | **45,75** | **59,50** |
|  | **SD** |  | **0,90** | **0,219** | **1,13** | **46026,88** | **36,06** | **44,77** |
| **F6** | **HC** |  |  |  |  |  |  |  |
| P02671 | FGA | Fibrinogen alpha chain | 5,56 | -0,822 | 5,7 | 94973,04 | 72 | 91 |
| P02675 | FGB | Fibrinogen beta chain | 4,95 | -0,758 | 8,54 | 55928,15 | 47 | 56 |
| P01619 | IGKV3-20 | Ig kappa chain V-III region WOL | 4,75 | -0,130 | 4,85 | 12557,12 | 12 | 12 |
| P02787 | TF | Serotransferrin | 4,70 | -0,336 | 6,81 | 77063,89 | 62 | 81 |
| P01857 | IGHG1 | Ig gamma-1 chain C region | 4,43 | -0,428 | 8,46 | 36105,91 | 27 | 36 |
| P00738 | HP | Haptoglobin | 4,30 | -0,421 | 6,13 | 45205,31 | 37 | 50 |
| P01024 | C3 | Complement C3 | 3,91 | -0,320 | 6,02 | 187148,06 | 134 | 163 |
| P01834 | IGKC | Ig kappa chain C region | 3,56 | -0,537 | 6,11 | 11765,04 | 9 | 11 |
| F5H0Q5 | AHSG | Alpha-2-HS-glycoprotein | 3,24 | -0,190 | 5,43 | 39340,74 | 20 | 33 |
| H0YGH4 | A2M | Alpha-2-macroglobulin | 2,90 | -0,195 | 6,03 | 163291 | 129 | 169 |
| P00734 | F2 | Prothrombin | 2,57 | -0,539 | 5,63 | 70036,87 | 56 | 69 |
| P10909-4 | CLU | Clusterin | 2,38 | -0,665 | 5,88 | 52494,58 | 39 | 52 |
| P01876 | IGHA1 | Ig alpha-1 chain C region | 2,37 | -0,206 | 6,08 | 37654,65 | 23 | 31 |
| P01766 | IGHV3-13 | Ig heavy chain V-III region BRO | 2,09 | 0,066 | 6,54 | 12506,22 | 13 | 14 |
| P04004 | VTN | Vitronectin | 2,01 | -0,723 | 5,55 | 54305,59 | 56 | 65 |
| P0CG05 | IGLC2 | Ig lambda-2 chain C regions | 1,93 | -0,444 | 6,91 | 11293,57 | 8 | 10 |
|  | **Average** |  | **3,48** | **-0,416** | **6,29** | **60104,36** | **46,50** | **58,94** |
|  | **SD** |  | **1,20** | **0,251** | **1,00** | **51301,20** | **38,74** | **48,74** |
| **F6** | **SC** |  |  |  |  |  |  |  |
| P02675 | FGB | Fibrinogen beta chain | 6,03 | -0,758 | 8,54 | 55928,15 | 47 | 56 |
| P01857 | IGHG1 | Ig gamma-1 chain C region | 5,84 | -0,428 | 8,46 | 36105,91 | 27 | 36 |
| P02787 | TF | Serotransferrin | 5,77 | -0,336 | 6,81 | 77063,89 | 62 | 81 |
| P02671 | FGA | Fibrinogen alpha chain | 5,63 | -0,822 | 5,7 | 94973,04 | 72 | 91 |
| P01009 | SERPINA1 | Alpha-1-antitrypsin | 5,63 | -0,183 | 5,37 | 46736,55 | 36 | 49 |
| P02647 | APOA1 | Apolipoprotein A-I | 5,58 | -0,717 | 5,56 | 30777,83 | 20 | 26 |
| P00738 | HP | Haptoglobin | 5,42 | -0,421 | 6,13 | 45205,31 | 37 | 50 |
| P01876 | IGHA1 | Ig alpha-1 chain C region | 5,28 | -0,206 | 6,08 | 37654,65 | 23 | 31 |
| H0YGH4 | A2M | Alpha-2-macroglobulin | 5,27 | -0,195 | 6,03 | 163291 | 129 | 169 |
| P01024 | C3 | Complement C3 | 4,82 | -0,320 | 6,02 | 187148,06 | 134 | 163 |
| P02763 | ORM1 | Alpha-1-acid glycoprotein 1 | 4,21 | -0,536 | 4,93 | 23511,56 | 24 | 27 |
| P06727 | APOA4 | Apolipoprotein A-IV | 4,16 | -0,796 | 5,28 | 45399,06 | 20 | 28 |
| P01859 | IGHG2 | Ig gamma-2 chain C region | 3,91 | -0,419 | 7,66 | 35900,64 | 28 | 36 |
| P01860 | IGHG3 | Ig gamma-3 chain C region | 3,81 | -0,511 | 8,23 | 41286,96 | 27 | 35 |
| P02790 | HPX | Hemopexin | 3,72 | -0,434 | 6,55 | 51676,37 | 55 | 74 |
| B0V2C8 | C4A | Complement C4-A | 3,53 | -0,252 | 6,66 | 192785,48 | 139 | 177 |
| P02656 | APOC3 | Apolipoprotein C-III | 3,53 | -0,086 | 5,23 | 10852,31 | 9 | 10 |
| P02679-2 | FGG | Fibrinogen gamma chain | 3,51 | -0,588 | 5,37 | 51511,66 | 55 | 67 |

| **F1 HC** | Average SD |  | 5,42  0,91 | -0,477 0,225 | 6,25  0,98 | 80008,28  112130,65 | 62,05  90,00 | 78,85  114,60 |
| --- | --- | --- | --- | --- | --- | --- | --- | --- |
|  |  |  |  |  |  |  |  |  |
| **F1 SC** | Average SD |  | 4,15  1,24 | -0,439 0,214 | 6,50  1,01 | 63855,90  54531,47 | 48,55  40,87 | 62,10  51,62 |
|  |  |  |  |  |  |  |  |  |

| **F2 HC** | Average SD |  | 5,20  0,84 | -0,439 0,208 | 6,30  0,97 | 98278,34  121408,55 | 75,10  95,45 | 95,50  121,35 |
| --- | --- | --- | --- | --- | --- | --- | --- | --- |
|  |  |  |  |  |  |  |  |  |
| **F2 SC** | Average SD |  | 4,41  1,23 | -0,436 0,216 | 6,60  1,08 | 63298,82  54742,99 | 48,75  40,81 | 62,15  51,61 |
|  |  |  |  |  |  |  |  |  |

| **All HC** | **Average SD** | **3,84**  **1,75** | **-0,415 0,229** | **6,35**  **1,02** | **87064,66**  **111169,11** | **67,51**  **87,57** | **85,71**  **111,40** |
| --- | --- | --- | --- | --- | --- | --- | --- |
|  |  |  |  |  |  |  |  |
|  | |  |  |  |  |  |  |
| **All SC** | **Average SD** | **4,50**  **1,06** | **-0,447 0,212** | **6,43**  **1,07** | **60155,44**  **49847,93** | **46,49**  **38,31** | **59,63**  **48,35** |
|  |  |  |  |  |  |  |  |
|  | |  |  |  |  |  |  |
| **Source** | **Average SD** | **4,26**  **1,15** | **-0,434 0,203** | **6,35**  **1,07** | **62320,15**  **48874,87** | **48,15**  **39,34** | **61,30**  **48,74** |
|  |  |  |  |  |  |  |  |

| P01834 | IGKC | Ig kappa chain C region | 3,51 | -0,537 | 6,11 | 11765,04 | 9 | 11 |
| --- | --- | --- | --- | --- | --- | --- | --- | --- |
| F5H0Q5 | AHSG | Alpha-2-HS-glycoprotein | 3,26 | -0,190 | 5,43 | 39340,74 | 20 | 33 |
|  | **Average** |  | **4,62** | **-0,437** | **6,31** | **63945,71** | **48,65** | **62,50** |
|  | **SD** |  | **0,98** | **0,221** | **1,11** | **54188,71** | **40,55** | **51,02** |
| **F7** | **HC** |  |  |  |  |  |  |  |
| P02671 | FGA | Fibrinogen alpha chain | 5,26 | -0,822 | 5,7 | 94973,04 | 72 | 91 |
| P01024 | C3 | Complement C3 | 4,17 | -0,320 | 6,02 | 187148,06 | 134 | 163 |
| P01857 | IGHG1 | Ig gamma-1 chain C region | 4,17 | -0,428 | 8,46 | 36105,91 | 27 | 36 |
| P02675 | FGB | Fibrinogen beta chain | 4,08 | -0,758 | 8,54 | 55928,15 | 47 | 56 |
| P01766 | IGHV3-13 | Ig heavy chain V-III region BRO | 3,15 | 0,066 | 6,54 | 12506,22 | 13 | 14 |
| P10909-4 | CLU | Clusterin | 3,07 | -0,665 | 5,88 | 52494,58 | 39 | 52 |
| P01834 | IGKC | Ig kappa chain C region | 3,04 | -0,537 | 6,11 | 11765,04 | 9 | 11 |
| P00738 | HP | Haptoglobin | 2,89 | -0,421 | 6,13 | 45205,31 | 37 | 50 |
| P02787 | TF | Serotransferrin | 2,61 | -0,336 | 6,81 | 77063,89 | 62 | 81 |
| P01619 | IGKV3-20 | Ig kappa chain V-III region WOL | 2,55 | -0,130 | 4,85 | 12557,12 | 12 | 12 |
| H0YGH4 | A2M | Alpha-2-macroglobulin | 2,05 | -0,195 | 6,03 | 163291 | 129 | 169 |
| B0V2C8 | C4A | Complement C4-A | 1,87 | -0,252 | 6,66 | 192785,48 | 139 | 177 |
| P0CG05 | IGLC2 | Ig lambda-2 chain C regions | 1,76 | -0,444 | 6,91 | 11293,57 | 8 | 10 |
| F5H0Q5 | AHSG | Alpha-2-HS-glycoprotein | 1,37 | -0,190 | 5,43 | 39340,74 | 20 | 33 |
| P01764 | IGHV3-23 | Ig heavy chain V-III region TIL | 1,04 | 0,073 | 8,49 | 12582,31 | 14 | 14 |
| P01859 | IGHG2 | Ig gamma-2 chain C region | 1,01 | -0,419 | 7,66 | 35900,64 | 28 | 36 |
| P04114 | APOB | Apolipoprotein B-100 | 1,00 | -0,296 | 6,58 | 515604,73 | 414 | 528 |
| P02751-14 | FN1 | Fibronectin | 0,91 | -0,514 | 5,31 | 272320,36 | 198 | 249 |
| P04004 | VTN | Vitronectin | 0,50 | -0,723 | 5,55 | 54305,59 | 56 | 65 |
| P06312 | IGKV4-1 | Ig kappa chain V-IV region Len | 0,39 | -0,143 | 5,09 | 13380,05 | 15 | 15 |
|  | **Average** |  | **2,35** | **-0,373** | **6,44** | **94827,59** | **73,65** | **93,10** |
|  | **SD** |  | **1,39** | **0,252** | **1,11** | **123524,62** | **96,27** | **122,68** |
| **F7** | **SC** |  |  |  |  |  |  |  |
| P02647 | APOA1 | Apolipoprotein A-I | 6,02 | -0,717 | 5,56 | 30777,83 | 20 | 26 |
| P02787 | TF | Serotransferrin | 5,65 | -0,336 | 6,81 | 77063,89 | 62 | 81 |
| P01876 | IGHA1 | Ig alpha-1 chain C region | 5,64 | -0,206 | 6,08 | 37654,65 | 23 | 31 |
| P02671 | FGA | Fibrinogen alpha chain | 5,40 | -0,822 | 5,7 | 94973,04 | 72 | 91 |
| P01009 | SERPINA1 | Alpha-1-antitrypsin | 5,24 | -0,183 | 5,37 | 46736,55 | 36 | 49 |
| P01024 | C3 | Complement C3 | 5,12 | -0,320 | 6,02 | 187148,06 | 134 | 163 |
| P02675 | FGB | Fibrinogen beta chain | 5,04 | -0,758 | 8,54 | 55928,15 | 47 | 56 |
| P01857 | IGHG1 | Ig gamma-1 chain C region | 4,58 | -0,428 | 8,46 | 36105,91 | 27 | 36 |
| P0CG05 | IGLC2 | Ig lambda-2 chain C regions | 4,54 | -0,444 | 6,91 | 11293,57 | 8 | 10 |
| H0YGH4 | A2M | Alpha-2-macroglobulin | 4,49 | -0,195 | 6,03 | 163291 | 129 | 169 |
| P02763 | ORM1 | Alpha-1-acid glycoprotein 1 | 4,25 | -0,536 | 4,93 | 23511,56 | 24 | 27 |
| P01834 | IGKC | Ig kappa chain C region | 4,19 | -0,537 | 6,11 | 11765,04 | 9 | 11 |
| P06727 | APOA4 | Apolipoprotein A-IV | 4,06 | -0,796 | 5,28 | 45399,06 | 20 | 28 |
| F5H0Q5 | AHSG | Alpha-2-HS-glycoprotein | 3,96 | -0,190 | 5,43 | 39340,74 | 20 | 33 |
| P02679-2 | FGG | Fibrinogen gamma chain | 3,85 | -0,588 | 5,37 | 51511,66 | 55 | 67 |
| P02656 | APOC3 | Apolipoprotein C-III | 3,73 | -0,086 | 5,23 | 10852,31 | 9 | 10 |
| P01619 | IGKV3-20 | Ig kappa chain V-III region WOL | 3,66 | -0,130 | 4,85 | 12557,12 | 12 | 12 |
| P01766 | IGHV3-13 | Ig heavy chain V-III region BRO | 3,48 | 0,066 | 6,54 | 12506,22 | 13 | 14 |
| P02790 | HPX | Hemopexin | 3,36 | -0,434 | 6,55 | 51676,37 | 55 | 74 |
| P01860 | IGHG3 | Ig gamma-3 chain C region | 3,14 | -0,511 | 8,23 | 41286,96 | 27 | 35 |
|  | **Average** |  | **4,47** | **-0,408** | **6,20** | **52068,98** | **40,10** | **51,15** |
|  | **SD** |  | **0,84** | **0,254** | **1,12** | **47734,15** | **36,51** | **46,21** |
|  | |  | **Fold** | **GRAVY** | **pI** | **MW** | **F+Y+W** | **F+Y+W+H** |
| **All HC+SC Average** | |  | **4,18** | **-0,431** | **6,39** | **72793,49** | **56,36** | **71,85** |
| **SD** | |  | **1,46** | **0,219** | **1,04** | **84875,06** | **66,62** | **84,55** |

| **F3 HC** | Average SD | 5,28  0,95 | -0,450 0,217 | 6,28  0,98 | 80345,20  112012,63 | 62,20  89,90 | 79,80  114,26 |
| --- | --- | --- | --- | --- | --- | --- | --- |
|  |  |  |  |  |  |  |  |
| **F3 SC** | Average SD | 4,05  1,02 | -0,477 0,205 | 6,52  1,09 | 60218,69  47872,95 | 46,41  36,78 | 59,59  46,15 |
|  |  |  |  |  |  |  |  |
| **F4 HC** | Average SD | 2,31  1,30 | -0,391 0,244 | 6,56  1,16 | 91856,84  120654,22 | 72,45  94,55 | 91,50  120,40 |
|  |  |  |  |  |  |  |  |
| **F4 SC** | Average SD | 4,79  0,96 | -0,465 0,174 | 6,49  1,06 | 57328,87  49345,16 | 47,20  41,19 | 60,40  52,61 |
|  |  |  |  |  |  |  |  |
| **F5 HC** | Average SD | 2,75  1,31 | -0,361 0,215 | 6,33  1,01 | 98639,95  122409,93 | 76,45  95,68 | 96,95  121,77 |
|  |  |  |  |  |  |  |  |
| **F5 SC** | Average SD | 4,95  0,90 | -0,473 0,219 | 6,40  1,13 | 60380,59  46026,88 | 45,75  36,06 | 59,50  44,77 |
|  |  |  |  |  |  |  |  |
| **F6 HC** | Average SD | 3,48  1,20 | -0,416 0,251 | 6,29  1,00 | 60104,36  51301,20 | 46,50  38,74 | 58,94  48,74 |
|  |  |  |  |  |  |  |  |
| **F6 SC** | Average SD | 4,62  0,98 | -0,437 0,221 | 6,31  1,11 | 63945,71  54188,71 | 48,65  40,55 | 62,50  51,02 |
|  |  |  |  |  |  |  |  |
| **F7 HC** | Average SD | 2,35  1,39 | -0,373 0,252 | 6,44  1,11 | 94827,59  123524,62 | 73,65  96,27 | 93,10  122,68 |
|  |  |  |  |  |  |  |  |
| **F7 SC** | Average SD | 4,47  0,84 | -0,408 0,254 | 6,20  1,12 | 52068,98  47734,15 | 40,10  36,51 | 51,15  46,21 |
|  |  |  |  |  |  |  |  |

**Supplementary File 5**

**Top 20 PLASMA vs VITREOUS**

| **PLASMA** | | **Fold** | **GRAVY** | **pI** | **MW** | **F+Y+W** | **F+Y+W+H VITREOUS** | | | | **Fold** | **GRAVY** | **pI** | **MW** | **F+Y+W** | **F+Y+W+H** |
| --- | --- | --- | --- | --- | --- | --- | --- | --- | --- | --- | --- | --- | --- | --- | --- | --- |
| **All HC+SC Average SD** | | **4,18**  **1,46** | **-0,431 0,219** | **6,39**  **1,04** | **72793,49**  **84875,06** | **56,36**  **66,62** | **71,85**  **84,55** |  | **All HC+SC Average SD** | | **25,96 4,72** | **-0,406 0,258** | **6,49**  **1,34** | **49278,20**  **33837,75** | **35,90**  **22,97** | **47,08**  **29,96** |
|  |  |  |  |  |  |  |  |  |  |  |  |  |  |  |  |  |
|  | |  |  |  |  |  |  |  |  | |  |  |  |  |  |  |
| **All HC** | **Average SD** | **3,84**  **1,75** | **-0,415 0,229** | **6,35**  **1,02** | **87064,66**  **111169,11** | **67,51**  **87,57** | **85,71**  **111,40** |  | **All HC** | **Average SD** | **22,91 5,33** | **-0,370 0,240** | **6,46**  **1,58** | **54012,01**  **39585,59** | **39,42**  **28,61** | **51,18**  **36,83** |
|  |  |  |  |  |  |  |  |  |  |  |  |  |  |  |  |  |
|  | |  |  |  |  |  |  |  |  | |  |  |  |  |  |  |
| **All SC** | **Average SD** | **4,50**  **1,06** | **-0,447 0,212** | **6,43**  **1,07** | **60155,44**  **49847,93** | **46,49**  **38,31** | **59,63**  **48,35** |  | **All SC** | **Average SD** | **28,59 2,18** | **-0,436 0,268** | **6,52**  **1,13** | **45642,01**  **27700,44** | **32,86**  **16,24** | **43,44**  **22,09** |
|  |  |  |  |  |  |  |  |  |  |  |  |  |  |  |  |  |
|  | |  |  |  |  |  |  |  |  | |  |  |  |  |  |  |
| **Source** | **Average SD** | **4,26**  **1,15** | **-0,434 0,203** | **6,35**  **1,07** | **62320,15**  **48874,87** | **48,15**  **39,34** | **61,30**  **48,74** |  | **Source** | **Average SD** | **27,69 0,93** | **-0,428 0,278** | **6,53**  **1,16** | **45361,11**  **31011,87** | **34,35**  **20,29** | **45,65**  **26,66** |
|  |  |  |  |  |  |  |  |  |  |  |  |  |  |  |  |  |
|  | |  |  |  |  |  |  |  |  | |  |  |  |  |  |  |
| F1 HC | Average SD | 5,42  0,91 | -0,477 0,225 | 6,25  0,98 | 80008,28  112130,65 | 62,05  90,00 | 78,85  114,60 |  |  |  |  |  |  |  |  |  |
|  |  |  |  |  |  |  |  |  |  |  |  |  |  |  |  |  |
| F1 SC | Average SD | 4,15  1,24 | -0,439 0,214 | 6,50  1,01 | 63855,90  54531,47 | 48,55  40,87 | 62,10  51,62 |  |  |  |  |  |  |  |  |  |
|  |  |  |  |  |  |  |  |  |  |  |  |  |  |  |  |  |
|  | |  |  |  |  |  |  |  |  |  |  |  |  |  |  |  |
| F2 HC | Average SD | 5,20  0,84 | -0,439 0,208 | 6,30  0,97 | 98278,34  121408,55 | 75,10  95,45 | 95,50  121,35 |  | F2 HC | Average SD | 18,77 5,24 | -0,333 0,258 | 6,70  1,77 | 56228,26  29059,52 | 39,58  19,17 | 51,68  25,50 |
|  |  |  |  |  |  |  |  |  |  |  |  |  |  |  |  |  |
| F2 SC | Average SD | 4,41  1,23 | -0,436 0,216 | 6,60  1,08 | 63298,82  54742,99 | 48,75  40,81 | 62,15  51,61 |  | F2 SC | Average SD | 27,41 1,24 | -0,415 0,260 | 6,67  1,21 | 44391,36  27598,04 | 32,65  16,53 | 42,80  22,55 |
|  |  |  |  |  |  |  |  |  |  |  |  |  |  |  |  |  |
|  | |  |  |  |  |  |  |  |  | |  |  |  |  |  |  |
| F3 HC | Average SD | 5,28  0,95 | -0,450 0,217 | 6,28  0,98 | 80345,20  112012,63 | 62,20  89,90 | 79,80  114,26 |  |  |  |  |  |  |  |  |  |
|  |  |  |  |  |  |  |  |  |  |  |  |  |  |  |  |  |
| F3 SC | Average SD | 4,05  1,02 | -0,477 0,205 | 6,52  1,09 | 60218,69  47872,95 | 46,41  36,78 | 59,59  46,15 |  |  |  |  |  |  |  |  |  |
|  |  |  |  |  |  |  |  |  |  |  |  |  |  |  |  |  |
|  | |  |  |  |  |  |  |  |  |  |  |  |  |  |  |  |
| F4 HC | Average SD | 2,31  1,30 | -0,391 0,244 | 6,56  1,16 | 91856,84  120654,22 | 72,45  94,55 | 91,50  120,40 |  | F4 HC | Average SD | 22,23 6,52 | -0,375 0,235 | 6,33  1,48 | 68694,53  62822,56 | 52,37  44,71 | 67,63  57,14 |
|  |  |  |  |  |  |  |  |  |  |  |  |  |  |  |  |  |
| F4 SC | Average SD | 4,79  0,96 | -0,465 0,174 | 6,49  1,06 | 57328,87  49345,16 | 47,20  41,19 | 60,40  52,61 |  | F4 SC | Average SD | 31,80 0,95 | -0,488 0,295 | 6,35  1,09 | 46946,83  30437,77 | 32,70  16,51 | 43,55  22,44 |
|  |  |  |  |  |  |  |  |  |  |  |  |  |  |  |  |  |
|  | |  |  |  |  |  |  |  |  | |  |  |  |  |  |  |
| F5 HC | Average SD | 2,75  1,31 | -0,361 0,215 | 6,33  1,01 | 98639,95  122409,93 | 76,45  95,68 | 96,95  121,77 |  |  |  |  |  |  |  |  |  |
|  |  |  |  |  |  |  |  |  |  |  |  |  |  |  |  |  |
| F5 SC | Average SD | 4,95  0,90 | -0,473 0,219 | 6,40  1,13 | 60380,59  46026,88 | 45,75  36,06 | 59,50  44,77 |  |  |  |  |  |  |  |  |  |
|  |  |  |  |  |  |  |  |  |  |  |  |  |  |  |  |  |
|  | |  |  |  |  |  |  |  |  |  |  |  |  |  |  |  |
| F6 HC | Average SD | 3,48  1,20 | -0,416 0,251 | 6,29  1,00 | 60104,36  51301,20 | 46,50  38,74 | 58,94  48,74 |  | F6 HC | Average SD | 26,98 1,21 | -0,377 0,241 | 6,42  1,50 | 48219,98  30650,82 | 34,60  21,54 | 45,05  28,83 |
|  |  |  |  |  |  |  |  |  |  |  |  |  |  |  |  |  |
| F6 SC | Average SD | 4,62  0,98 | -0,437 0,221 | 6,31  1,11 | 63945,71  54188,71 | 48,65  40,55 | 62,50  51,02 |  | F6 SC | Average SD | 27,57 1,17 | -0,421 0,265 | 6,53  1,14 | 45614,92  27375,74 | 33,05  16,59 | 43,70  22,54 |
|  |  |  |  |  |  |  |  |  |  |  |  |  |  |  |  |  |
|  | |  |  |  |  |  |  |  |  | |  |  |  |  |  |  |
| F7 HC | Average SD | 2,35  1,39 | -0,373 0,252 | 6,44  1,11 | 94827,59  123524,62 | 73,65  96,27 | 93,10  122,68 |  | F7 HC | Average SD | 23,68 3,14 | -0,393 0,239 | 6,38  1,64 | 43750,21  22482,56 | 31,80  18,91 | 41,20  23,25 |
|  |  |  |  |  |  |  |  |  |  |  |  |  |  |  |  |  |
| F7 SC | Average SD | 4,47  0,84 | -0,408 0,254 | 6,20  1,12 | 52068,98  47734,15 | 40,10  36,51 | 51,15  46,21 |  | F7 SC | Average SD | 27,57 1,21 | -0,421 0,265 | 6,53  1,14 | 45614,92  27375,74 | 33,05  16,59 | 43,70  22,54 |
|  |  |  |  |  |  |  |  |  |  |  |  |  |  |  |  |  |

**Supplementary File 6**

**Common gene subsets PLASMA and VITREOUS**

| **Common gene subset PLASMA** | | | | |  |  | |  | |
| --- | --- | --- | --- | --- | --- | --- | --- | --- | --- |
| **22** |  |  | **GRAVY** | | **pI** | **MW** | **F+Y+W** |  | **F+Y+W+H** |
| P01834 | IGKC | Immunoglobulin kappa constant (Ig kappa chain C region) |  | -0,537 | 6,11 | 11765,04 |  | 9 | 11 |
| P02671 | FGA | Fibrinogen alpha chain [Cleaved into: Fibrinopeptide A; Fi | b | -0,822 | 5,7 | 94973,04 |  | 72 | 91 |
| P02787 | TF | Serotransferrin (Transferrin) (Beta-1 metal-binding globuli |  | -0,336 | 6,81 | 77063,89 |  | 62 | 81 |
| P0DOY2 | IGLC2 | Immunoglobulin lambda constant 2 (Ig lambda chain C re |  | -0,444 | 6,91 | 11293,57 |  | 8 | 10 |
| P02647 | APOA1 | Apolipoprotein A-I (Apo-AI) (ApoA-I) (Apolipoprotein A1) [ | C | -0,717 | 5,56 | 30777,83 |  | 20 | 26 |
| P01876 | IGHA1 | Immunoglobulin heavy constant alpha 1 (Ig alpha-1 chain |  | -0,206 | 6,08 | 37654,65 |  | 23 | 31 |
| P01009 | SERPINA1 | Alpha-1-antitrypsin (Alpha-1 protease inhibitor) (Alpha-1- | a | -0,183 | 5,37 | 46736,55 |  | 36 | 49 |
| P02679 | FGG | Fibrinogen gamma chain |  | -0,588 | 5,37 | 51511,66 |  | 55 | 67 |
| P06727 | APOA4 | Apolipoprotein A-IV (Apo-AIV) (ApoA-IV) (Apolipoprotein A |  | -0,796 | 5,28 | 45399,06 |  | 20 | 28 |
| P01857 | IGHG1 | Immunoglobulin heavy constant gamma 1 (Ig gamma-1 ch |  | -0,428 | 8,46 | 36105,91 |  | 27 | 36 |
| P01859 | IGHG2 | Immunoglobulin heavy constant gamma 2 (Ig gamma-2 ch |  | -0,419 | 7,66 | 35900,64 |  | 28 | 36 |
| P01023 | A2M | Alpha-2-macroglobulin (Alpha-2-M) (C3 and PZP-like alph | a | -0,195 | 6,03 | 163291 |  | 129 | 169 |
| P01024 | C3 | Complement C3 (C3 and PZP-like alpha-2-macroglobulin d |  | -0,320 | 6,02 | 187148,06 |  | 134 | 163 |
| P02675 | FGB | Fibrinogen beta chain [Cleaved into: Fibrinopeptide B; Fib |  | -0,758 | 8,54 | 55928,15 |  | 47 | 56 |
| P00738 | HP | Haptoglobin (Zonulin) [Cleaved into: Haptoglobin alpha c | h | -0,421 | 6,13 | 45205,31 |  | 37 | 50 |
| P0C0L4 | C4A | Complement C4-A (Acidic complement C4) (C3 and PZP-lik |  | -0,252 | 6,66 | 192785,48 |  | 139 | 177 |
| P02790 | HPX | Hemopexin (Beta-1B-glycoprotein) |  | -0,434 | 6,55 | 51676,37 |  | 55 | 74 |
| P01860 | IGHG3 | Immunoglobulin heavy constant gamma 3 (HDC) (Heavy c | h | -0,511 | 8,23 | 41286,96 |  | 27 | 35 |
| P0CF74 | IGLC6 | Immunoglobulin lambda constant 6 (Ig lambda-6 chain C |  | -0,467 | 6,91 | 11276,54 |  | 8 | 10 |
| P19823 | ITIH2 | Inter-alpha-trypsin inhibitor heavy chain H2 (ITI heavy cha |  | -0,293 | 6,4 | 106463,47 |  | 87 | 110 |
| P02765 | AHSG | Alpha-2-HS-glycoprotein (Alpha-2-Z-globulin) (Ba-alpha-2- |  | -0,190 | 5,43 | 39340,74 |  | 20 | 33 |
|  | Ig | Immunoglobulin variable |  |  |  |  |  |  |  |
|  |  | **Average** |  | **-0,444** | **6,49** | **65408,76** |  | **49,67** | **63,95** |
|  |  | **SD** |  | **0,202** | **1,01** | **54151,26** |  | **41,12** | **51,56** |
| **Common genes to all formulations PLASMA** | | | | |  |  | |  | |
| **7** |  |  | **GRAVY** | | **pI** | **MW** | **F+Y+W** |  | **F+Y+W+H** |
| P02671 | FGA | Fibrinogen alpha chain [Cleaved into: Fibrinopeptide A; Fi | b -0,822 | | 5,7 | 94973,04 |  | 72 | 91 |
| P01834 | IGKC | Immunoglobulin kappa constant (Ig kappa chain C region) | -0,537 | | 6,11 | 11765,04 |  | 9 | 11 |
| P02787 | TF | Serotransferrin (Transferrin) (Beta-1 metal-binding globuli | -0,336 | | 6,81 | 77063,89 |  | 62 | 81 |
| P01857 | IGHG1 | Immunoglobulin heavy constant gamma 1 (Ig gamma-1 ch | -0,428 | | 8,46 | 36105,91 |  | 27 | 36 |
| P01023 | A2M | Alpha-2-macroglobulin (Alpha-2-M) (C3 and PZP-like alph | a -0,195 | | 6,03 | 163291,00 |  | 129 | 169 |
| P01024 | C3 | Complement C3 (C3 and PZP-like alpha-2-macroglobulin d | -0,320 | | 6,02 | 187148,06 |  | 134 | 163 |
| P02675 | FGB | Fibrinogen beta chain [Cleaved into: Fibrinopeptide B; Fib | -0,758 | | 8,54 | 55928,15 |  | 47 | 56 |
|  |  | **Average** | **-0,485** | | **6,81** | **106713,37** |  | **68,57** | **86,71** |
|  |  | **SD** | **0,234** | | **1,20** | **70455,55** |  | **47,84** | **60,40** |

All

FGA IGKC TF IGHG1 A2M C3 FGB

7

Common subset Plasma

IGHG3 IGLC2 APOA1 IGHA1 FGG IGHG2 AHSG HP

8

Common subset

HPX Ig IGLC6 SERPINA1 C4A APOA4 ITIH2

7

Plasma enriched

F2 GC CFH APOC3 A1BG

5


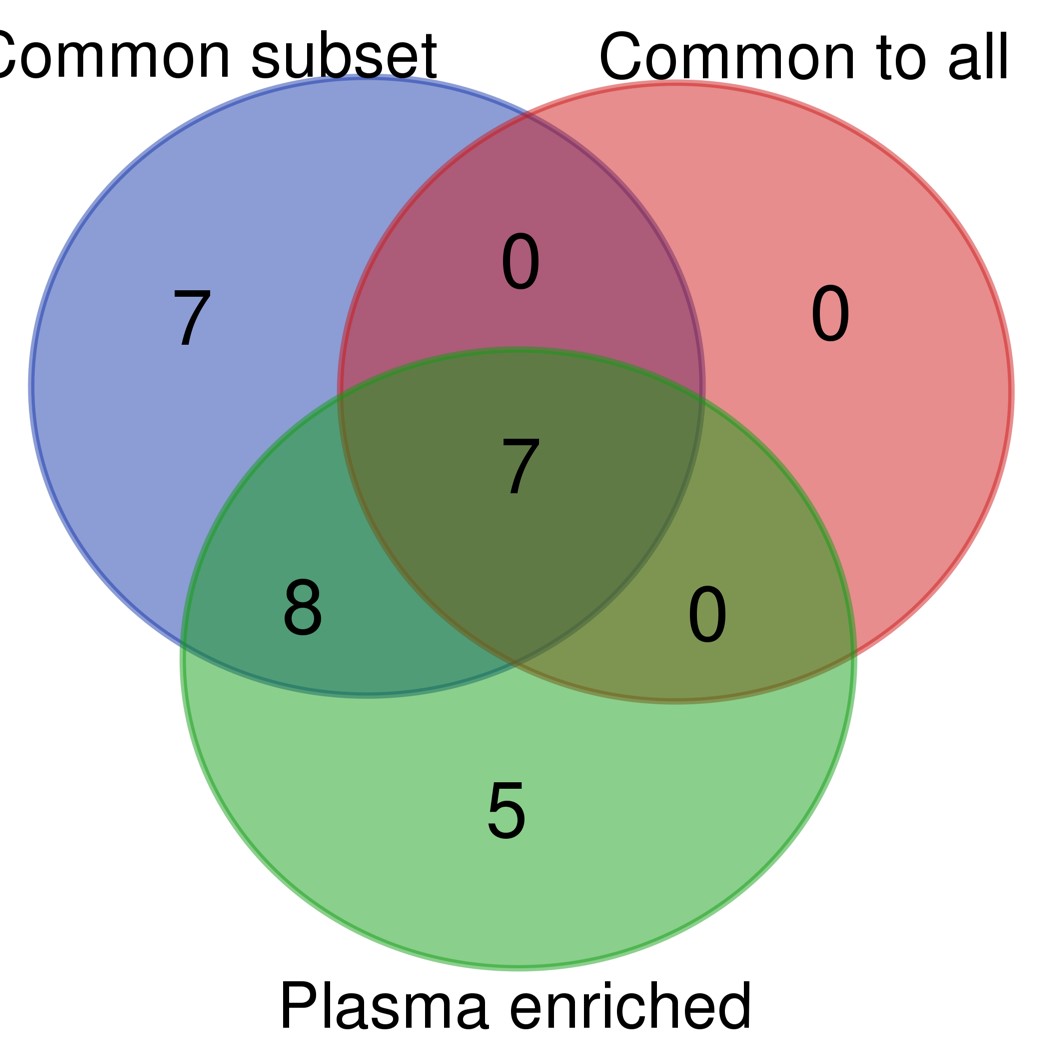


| **Common subset VITREOUS** | | | | |  |  | |  | |
| --- | --- | --- | --- | --- | --- | --- | --- | --- | --- |
| **14** |  |  | **GRAVY** | | **pI** | **MW** | **F+Y+W** |  | **F+Y+W+H** |
| P02489 | CRYAA | Alpha-crystallin A chain (Heat shock protein beta-4) (HspB | 4 | -0,490 | 5,77 | 19909,32 |  | 21 | 28 |
| P02788 | LTF | Lactotransferrin (Lactoferrin) (EC 3.4.21.-) (Growth-inhibi | t | -0,337 | 8,5 | 78181,95 |  | 63 | 72 |
| P60709 | ACTB | Actin, cytoplasmic 1 (Beta-actin) [Cleaved into: Actin, cyto |  | -0,200 | 5,29 | 41736,73 |  | 32 | 41 |
| P41219 | PRPH | Peripherin (Neurofilament 4) |  | -0,778 | 5,37 | 53650,89 |  | 24 | 33 |
| P43320 | CRYBB2 | Beta-crystallin B2 (Beta-B2 crystallin) (Beta-crystallin Bp) |  | -0,890 | 6,5 | 23379,94 |  | 22 | 31 |
| P10745 | RBP3 | Retinol-binding protein 3 (Interphotoreceptor retinoid-bi |  | 0,041 | 4,98 | 135362,66 |  | 84 | 121 |
| P04406 | GAPDH | Glyceraldehyde-3-phosphate dehydrogenase (GAPDH) (EC |  | -0,108 | 8,57 | 36053,21 |  | 26 | 36 |
| P10909 | CLU | Clusterin (Aging-associated gene 4 protein) (Apolipoprotei |  | -0,665 | 5,88 | 52494,58 |  | 39 | 52 |
| P63261 | ACTG1 | Actin, cytoplasmic 2 (Gamma-actin) [Cleaved into: Actin, c |  | -0,199 | 5,31 | 41792,84 |  | 32 | 41 |
| P02511 | CRYAB | Alpha-crystallin B chain (Alpha(B)-crystallin) (Heat shock p |  | -0,531 | 6,76 | 20158,91 |  | 18 | 27 |
| P12277 | CKB | Creatine kinase B-type (EC 2.7.3.2) (Brain creatine kinase) ( |  | -0,421 | 5,34 | 42644,28 |  | 30 | 43 |
| P06733 | ENO1 | Alpha-enolase (EC 4.2.1.11) (2-phospho-D-glycerate hydro |  | -0,221 | 7,01 | 47168,96 |  | 31 | 37 |
| P14618 | PKM | Pyruvate kinase PKM (EC 2.7.1.40) (Cytosolic thyroid horm |  | -0,128 | 7,96 | 57936,89 |  | 29 | 41 |
| P02768 | ALB | Serum albumin |  | -0,354 | 5,92 | 69366,68 |  | 56 | 72 |
| P41222 | PTGDS | Prostaglandin-H2 D-isomerase (EC 5.3.99.2) (Beta-trace pr |  | -0,325 | 7,66 | 21028,82 |  | 20 | 23 |
|  |  | **Average** |  | **-0,374** | **6,45** | **49391,11** |  | **35,13** | **46,53** |
|  |  | **SD** |  | **0,260** | **1,23** | **29603,61** |  | **18,55** | **25,19** |
| **Common to all formulations VITREOUS** | | | |  |  |  | |  | |
| **3** |  |  |  |  |  |  |  |  |  |
| P02489 | CRYAA | Alpha-crystallin A chain (Heat shock protein beta-4) (HspB | 4 | -0,490 | 5,77 | 19909,32 |  | 21 | 28 |
| P02788 | LTF | Lactotransferrin (Lactoferrin) (EC 3.4.21.-) (Growth-inhibi | t | -0,337 | 8,50 | 78181,95 |  | 63 | 72 |
| P43320 | CRYBB2 | Beta-crystallin B2 (Beta-B2 crystallin) (Beta-crystallin Bp) |  | -0,890 | 6,50 | 23379,94 |  | 22 | 31 |
|  |  | **Average** |  | **-0,572** | **6,92** | **40490,40** |  | **35,33** | **43,67** |
|  |  | **SD** |  | **0,286** | **1,41** | **32687,93** |  | **23,97** | **24,58** |

All

CRYAA LTF CRYBB2

3

Common subset Vitreous e

CKB PRPH ENO1 CLU PKM ALB RBP3 CRYAB GAPDH PTGDS

10

Common subset

ACTB ACTG1

2

Vitreous enriched

CRYBA4 CRYBA1 ALDOA CRYBB3 HBB TUBB1 CRYBA2

7


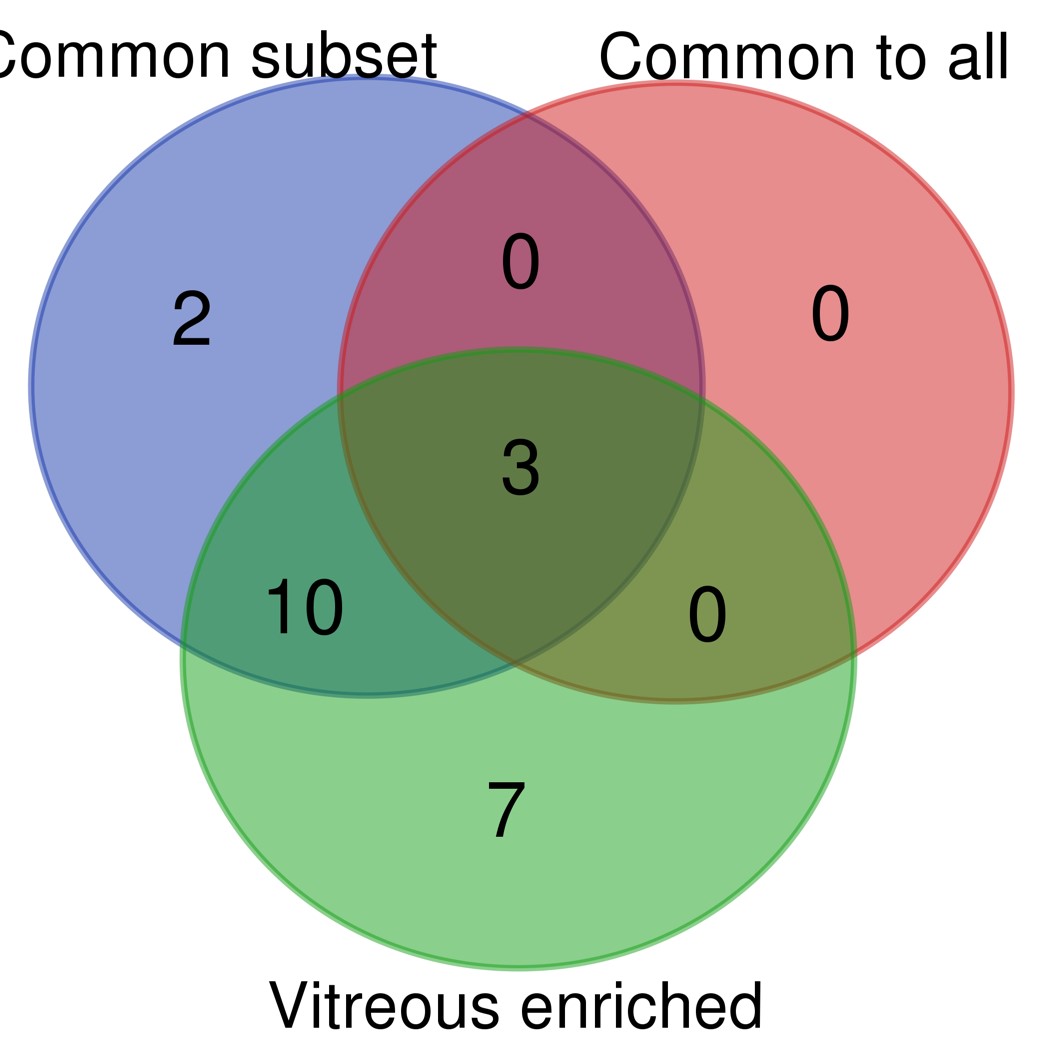

Supplement: Supplementary file 1 [file pharmaceutics-12-00763-s001.zip › pharmaceutics-874302-supplementary/Supplementary Information and Files 3-6 combined.docx]
